# TyCHE enables time-resolved lineage tracing of heterogeneously-evolving populations

**DOI:** 10.1101/2025.10.21.683591

**Published:** 2025-10-22

**Authors:** Jessie J. Fielding, Sherry Wu, Hunter J. Melton, Nic Fisk, Louis du Plessis, Kenneth B. Hoehn

**Affiliations:** 1 Department of Biomedical Data Science, Geisel School of Medicine at Dartmouth, Hanover, NH; 2 Department of Cell and Molecular Biology, University of Rhode Island, Kingston, RI; 3 Department of Biosystems Science and Engineering, ETH Zurich, Basel, Switzerland; 4 Swiss Institute of Bioinformatics, Lausanne, Vaud, Switzerland; 5 Dartmouth Cancer Center, Geisel School of Medicine at Dartmouth, Hanover, NH

## Abstract

Phylogenetic methods for cell lineage tracing have driven significant insights into organismal development, immune responses, and tumor evolution. While most methods estimate mutation trees, time-resolved lineage trees are more interpretable and could relate cellular migration and differentiation to perturbations like vaccines and drug treatments. However, somatic mutation rates can vary dramatically by cell type, significantly biasing existing methods. We introduce TyCHE (Type-linked Clocks for Heterogeneous Evolution), a Bayesian phylogenetics package that infers time-resolved phylogenies of populations with distinct evolutionary rates. We demonstrate that TyCHE improves tree accuracy using a new simulation package SimBLE (Simulator of B cell Lineage Evolution). We use TyCHE to infer patterns of memory B cell formation during HIV infection, dynamics of recall germinal centers following influenza vaccination, temporal evolution of a glioma tumor lineage, and progression of a bacterial lung infection. TyCHE and SimBLE are available as open-source software compatible with the BEAST2 and Immcantation ecosystems.

## Introduction

Human diseases are fundamentally shaped by cell proliferation, differentiation into subtypes, and migration across tissues. Phylogenetic methods for cellular lineage tracing have recently driven significant insights into development and disease.^1–6^ These methods either use mutations from implanted dynamic lineage recorders or naturally-occurring somatic mutations.^7–10^ While most phylogenetic methods estimate genetic distance trees in which branch lengths represent mutations per site, some approaches estimate time trees in which branch lengths represent calendar time.^11^ Time trees have significant advantages because they provide calendar date estimates for evolutionary events and can therefore relate them to external perturbations. For example, time trees have been used to identify the ignition date of the HIV-1 pandemic^12,13^ and the spread of SARS-CoV-2 globally.^14,15^ Furthermore, time trees reflect past population dynamics^16^ and can be used to infer epidemiological parameters like migration history and changes in effective population size, for example quantifying the effect of containment measures during recent Ebola virus and SARS-CoV-2 epidemics.^17–19^ Applied to cell populations, similar approaches could be used to infer cell migration, differentiation, and proliferation in response to perturbations like vaccines, antibiotics, and chemotherapy.

A major challenge for inferring time trees for cell populations is that cellular evolution is highly heterogeneous. While many viruses evolve in a clock-like manner, somatic mutations in cells are often linked to “hypermutator” states. For example, B cells undergo periods of rapid somatic hypermutation during immune responses before becoming quiescent memory cells.^20^ Infectious bacterial populations, viruses, and tumors also frequently develop hypermutator phenotypes.^21–24^ Furthermore, artificial, dynamic lineage recorders are frequently designed to mutate only during distinct periods induced by, for example, doxycycline.^25,26^ Existing methods for estimating time trees typically assume the evolutionary rate is uncorrelated with phenotype, leading to biased inferences when applied to populations with distinct evolutionary rates.^11,27^

Here, we introduce TyCHE (Type-linked Clocks for Heterogeneous Evolution), which integrates with the BEAST2^28^ Bayesian phylogenetics platform and estimates accurate time trees of heterogeneously evolving populations. In contrast to existing methods, TyCHE simultaneously reconstructs ancestral cell type states and estimates the dates of internal tree nodes using separate molecular clock rates for each cell type. To validate TyCHE, we developed SimBLE (Simulator of B cell Lineage Evolution), which simulates heterogeneously evolving populations, including realistic B cell germinal center (GC) reactions. Using simulations, we demonstrate that TyCHE estimates time trees with more accurate dates, topologies, and reconstructed cell types than existing methods. Using empirical B cell receptor (BCR) sequences, we show that TyCHE can infer realistic patterns of B cell evolution and memory B cell formation during chronic HIV infection^29^ as well as realistic dynamics of recall GCs following repeated influenza vaccination.^30^ Further, we use TyCHE to reconstruct the temporal evolution of a hypermutating glioma tumor lineage, and the evolution of a *Pseudomonas aeruginosa* hypermutator lineage during an acute lung infection.^22,31^ TyCHE and SimBLE both interface with the R package Dowser for cellular phylogenetic analyses, which is part of the Immcantation suite.^32^

## Results

### TyCHE: Type-linked clocks for heterogeneous evolution

Inferring time-resolved phylogenies requires a clock model, which describes the relationship between evolution and time. A “strict clock” (SC) assumes a constant rate of evolution. Other models, such as the uncorrelated lognormally distributed (UCLD) relaxed clock, allow rates to vary.^27,33^ Bayesian time tree estimation typically uses Markov Chain Monte Carlo (MCMC), in which tree topologies, node heights (dates), and parameter values are iteratively proposed. Each proposal, the clock model converts branch lengths from calendar time to genetic distance, enabling computation of the sequence data likelihood.^34^ New topologies, node heights, and parameter values are accepted or rejected based on their posterior probabilities relative to the current state.^11,28^

**Figure 1:**
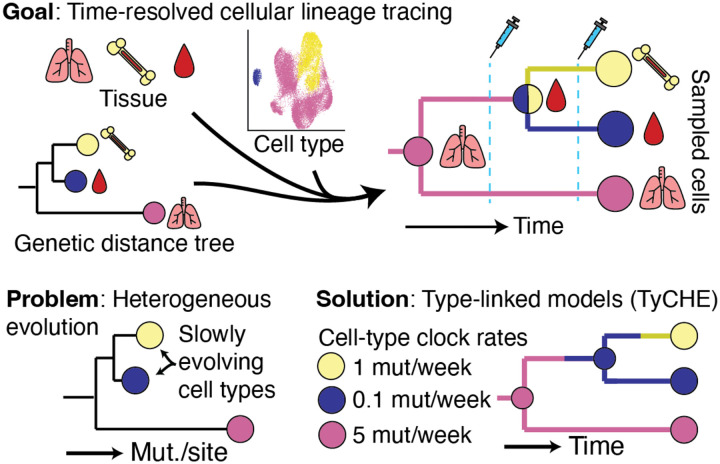
Graphical representation of TyCHE. Time-resolved cellular lineage tracing has the potential to unravel the dynamics of cellular evolution, migration, and differentiation. However, existing molecular clock models are inappropriate when evolutionary rates are linked to discrete cell types. TyCHE solves this problem using type-linked clock models.

In contrast to prior approaches that model trait values as independent of the clock rate, TyCHE links each cell type to a separate molecular clock rate which can be either fixed or estimated as a parameter (Fig. 1). These clock rates can be *a priori* estimated using root-to-tip regression or by fitting a strict clock model to pure cell populations.^11,35^ Each node is assigned a cell type and the genetic distance of each branch is calculated based on its length and the states of its parent and child nodes, which is accomplished by estimating the expected time spent in each cell type (Expected Occupancy, EO) or assuming transitions occur instantaneously after the parent node (Instant Switch, IS). The cell types at each node are modeled as continuous time Markov chains (CTMC). The cell type CTMC is parameterized by a transition rate matrix, which can be used to estimate the relative rates of cellular transitions and can incorporate key prior information such as irreversibility of cell differentiation events. Critically, in contrast to prior approaches that treat sequence data likelihood as conditionally independent of trait values, TyCHE explicitly samples cell type states at internal nodes using MCMC.^36^

### SimBLE: Simulating B cell evolution during germinal center reactions

B cell evolution is highly heterogeneous. B cells produce antibodies, whose structures are also expressed as membrane-bound BCRs. During immune responses, B cells can enter GC reactions, during which they upregulate enzymes that rapidly introduce mutations in a process called somatic hypermutation (SHM).^20^ GC B cells with affinity-improving mutations survive and proliferate, while others undergo apoptosis. GC B cells can differentiate into memory B cells (MBCs) which can be stimulated upon re-infection, or plasma cells which migrate to the bone marrow and release antibodies. In contrast to GC B cells, neither MBCs nor plasma cells undergo SHM, so time-resolved phylogenetic analyses of B cells must take these vastly different evolutionary rates into account.

**Figure 2:**
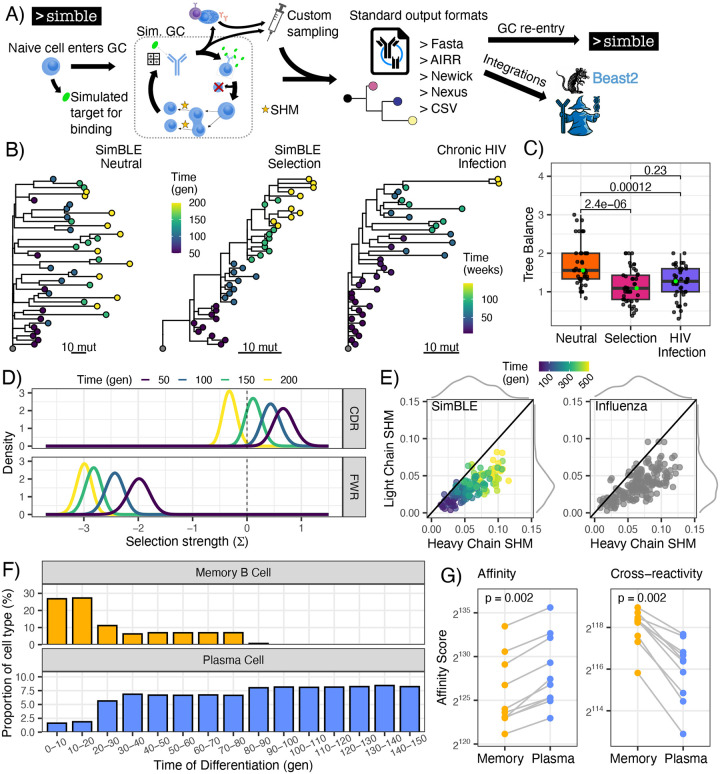
Simulating type-linked evolution using SimBLE. **A)** Graphical representation of the SimBLE algorithm. **B)** Estimated genetic distance trees from BCR sequences simulated under neutral and selective evolution, as well as previously published BCRs from chronic HIV infection.^49^ All lineages were downsampled to 40 sequences. Tips are colored by sample time in generations (gen) or weeks. **C)** Tree balance measured as maximum width over maximum depth^50,51^ for simulations of neutral and selective evolution for 200 generations, and published BCRs from measurably evolving lineages from an HIV-1 infected donor.^49^ Each dataset used 45 lineages downsampled to 40 sequences each. Trees from panel B are highlighted in green. **D)** Posterior distributions of selection scores from BASELINe applied to BCRs from 100 lineages sampled at specified timepoints under simulations of selection. Values less than zero indicate purifying selection while values greater than zero indicate diversifying selection. **E)** Heavy and light chain mutation frequency. Fifteen lineages were simulated under selection for each specified number of generations for a total of 150 lineages. Each dot represents the mean of a lineage. Marginal distributions are shown on each axis. Line shows equal SHM in both chains. BCRs sampled following influenza vaccination are also shown.^37,52^ **F)** Proportion of MBCs and plasma cells by time of differentiation from the GC in 100 simulated lineages with a migration rate of 5 cells/generation. **G)** Mean affinity to target sequence and mean affinity to 10 target sequences differing from the original by 5 heavy and 3 light chain AA substitutions (cross-reactivity). Ten lineages were simulated for 150 generations. Each dot shows the mean of that cell type in one lineage, with lines connecting cell types from the same lineage. P values were calculated using the Wilcoxon signed-rank test.

To benchmark TyCHE, we developed SimBLE, an agent-based simulator of heterogeneous evolution, including B cell evolution and differentiation in GCs (Fig. 2A). Briefly, a starting pair of BCR heavy and light chain sequences is randomly chosen from a dataset of naive single B cells.^37,38^ GC B cells mutate according to models of SHM targeting, and affinity is calculated based on similarity to a target amino acid sequence.^39,40^ B cells proliferate proportionally to their relative affinity. GC B cells differentiate into MBCs early on before shifting to primarily plasma cell production.^41^ SimBLE incorporates recent discoveries, including silencing of SHM during clonal bursts and a log-additive relationship between mutations and affinity.^42–44^

We compared BCRs simulated from SimBLE to empirical BCR sequences. Trees simulated under selection had imbalanced topologies similar to B cell trees from chronic HIV infection, while trees simulated under neutral evolution were more comparatively balanced (Fig. 2B–2C). BCRs simulated under selection showed diversifying selection in antigen-binding regions and purifying selection in structural framework regions (Fig. 2D).^45^ Selection shifted towards purifying selection over time, consistent with prior studies.^46–48^ These signatures of selection were absent in BCRs simulated under neutral evolution (Supplemental Fig. 1). Simulated BCRs had realistic SHM levels (5–10%) that were approximately twice as high in the heavy chain as the light chain, as shown previously (Fig. 2E).^37^ Site-wise substitution patterns were distinct among selection, neutral, and uniform neutral simulations (Supplemental Fig. 2). MBCs differentiated before plasma cells, closely matching prior estimates (Fig. 2F).^41^ Further, while plasma cells had higher affinity to their target sequence, MBCs showed higher affinity to similar non-target sequences, representing broader reactivity (Fig. 2G). These results confirm SimBLE produces realistic GC and non-GC BCR sequences.

### Simulation-based validation

**Figure 3:**
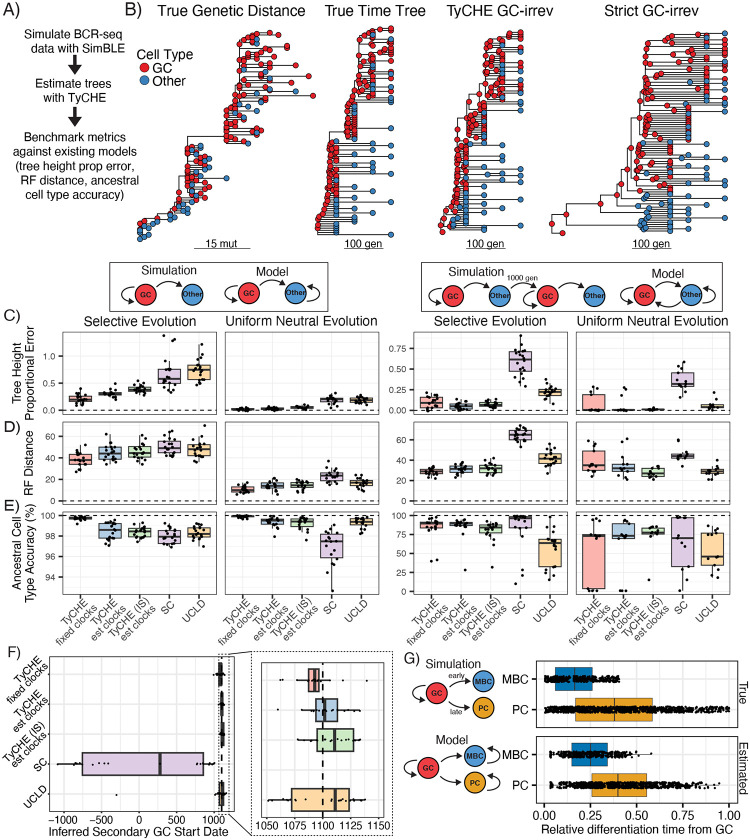
Simulation-based validation of TyCHE using SimBLE. **A)** Design of validation experiments. **B)** Example results from one simulated primary GC lineage. From left to right: the true genetic distance tree, the true time tree, the time tree estimated by TyCHE, and the time tree estimated using the SC model. All models assume switches from GC B cells to other B cells are irreversible (GC-irrev). **C)** Proportional error of tree height estimates. Each dot represents one clone (simulation replicate). **D)** Comparison of Robinson-Foulds distance from the true tree topology. To reduce noise, branches under 5 generations were collapsed. **E)** Mean accuracy of ancestral cell type prediction for the MRCA of each pairwise combination of tips. Eight clones were removed from uniform neutral GC recall simulations due to lack of convergence in at least one model. One further outlier clone was removed from uniform neutral recall GC results for clarity, but is shown in Supplemental Fig. 4. **F)** Inferred secondary GC start date for recall GC simulations. **G)** Estimated relative time of GC differentiation for MBCs and plasma cells compared to true values. Compare to Fig 2F.

We validated TyCHE’s performance using simulated sequences of primary and recall GCs from SimBLE. Specifically, we compared TyCHE’s EO and IS models to the existing SC and UCLD relaxed clock models (Fig. 3A). For TyCHE, the GC clock rate used the mean rate estimated by a strict clock model applied only to GC B cells in each simulation. The clock rate for non-GC B cells was set arbitrarily low at 10^−6^ SHM/site/generation. GC and non-GC clock rates were either fixed or estimated in conjunction with a normal prior distribution. For SC and UCLD models the clock rate, tree heights, and other parameters were estimated using all sequences simultaneously, as is standard. In simulated primary GC reactions, all models were constrained such that GC to non-GC transitions were irreversible. In simulated recall GC reactions, non-GC to GC transitions were allowed.

For each scenario, 20 lineages with 96 B cells sampled each were simulated. In primary GC reactions, 12 GC and 12 non-GC (MBC or plasma cell) B cells were sampled at generations 50, 100, 150, and 200 (Fig. 3A–B). In recall GC reaction simulations, 12 GC and 12 non-GC B cells were sampled at generations 50 and 100 during a primary GC reaction. An unsampled MBC from generation 100 was then randomly selected to initiate a secondary GC at generation 1100 (Supplemental Fig. 3). 12 GC and 12 non-GC B cells were sampled from this recall GC at generations 1150 and 1200. In primary and recall GCs both affinity-driven selection with biased SHM starting from a naive BCR sequence as well as neutral evolution with uniform substitution probabilities starting from a random sequence were simulated. Model performance was evaluated based on estimated tree height, i.e., the time from the most recent tip to the root, the Robinson-Foulds distance from the estimated tree topology to the true tree topology with branches under 5 generations collapsed, and the accuracy of the predicted cell types at the most recent common ancestor (MRCA) of each pairwise combination of tips.

In primary GC simulations, TyCHE’s EO model with fixed clock rates outperformed or was approximately equal to all other models evaluated (Fig. 3B–E, Supplemental Fig. 4). These results remained consistent when sampling GC and non-GC B cells at a 1:3 ratio (Supplemental Fig. 5). Results in recall GCs were more variable, but TyCHE’s EO model with estimated clock rates performed best overall (Fig. 3C–E, Supplemental Fig. 5). TyCHE’s IS model with estimated clock rates performed slightly worse than EO models, but was still competitive (Fig. 3C–E). TyCHE’s EO and IS models generally outperformed or tied with SC and UCLD models when comparing tree length, the sum of all branch lengths, excepting the EO models in uniform neutral simulations of recall GC simulations (Supplemental Fig. 6). In primary GCs, performance of all models was worse on sequences simulated under selection, possibly due to changing clock rates from increasing purifying selection over time (Fig. 2D).^46–48^ These results demonstrate that TyCHE outperforms existing models when clock rates are linked to cell type.

We next tested TyCHE’s ability to estimate the time of cell differentiation events. In simulations of recall GCs, TyCHE’s EO model with estimated clock rates best predicted the start of recall GC reactions at generation 1100 (Fig. 3F). Following that, we tested TyCHE’s ability to estimate MBC and plasma cell differentiation times from primary GCs. Because EO calculations are currently only developed for the two-state scenario, we used a three-state IS model with irreversible switches from the GC. After estimating tree topologies and node heights, we used the estimated date of each MBC and plasma cell’s most recent GC ancestor as the date of differentiation. TyCHE accurately reconstructed the relative timing of MBC vs plasma cell differentiation (Fig. 3G). These two results confirm TyCHE can accurately estimate the timing of differentiation events both into and out of GCs.

### Chronic GC reactions during HIV-1 infection

**Figure 4:**
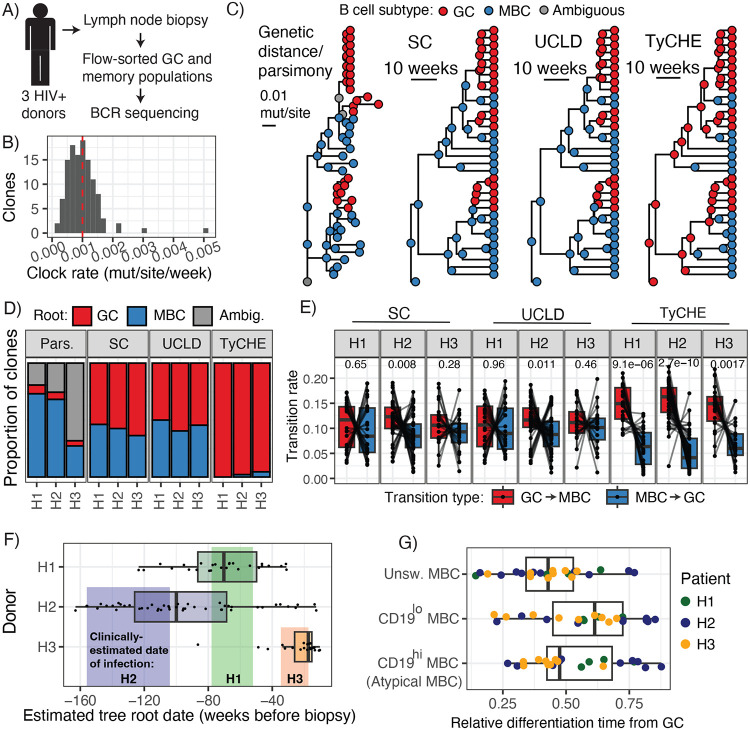
Time-resolved analysis of GC reactions in chronic HIV infection. **A)** Sampling strategy used in ^29^. **B)** Distribution of clock rate estimates using root-to-tip regression on a prior study of HIV-1 infection.^49^ Red line indicates the mean. **C)** Results from one lineage showing ancestral cell types predicted by maximum parsimony applied to a genetic distance tree, and time trees with ancestral cell type reconstructions from SC, UCLD, and the EO model in TyCHE. All models permitted reversible differentiation among GC B cells and MBCs for validation. **D)** Proportion of lineages predicting GC or MBC as the root state. **E)** Estimated relative transition rates from GC to MBC vs MBC to GC. P values shown above were computed with the Wilcoxon signed-rank test. **F)** Estimated root dates (GC initiation date) for each lineage analyzed using TyCHE. Each dot represents a lineage. Estimated date of infection ranges for each patient are shown in colored bands. **G)** Estimated relative time of differentiation from the GC for each MBC subtype. Each dot represents the average among cells of each type within a lineage.

HIV infection stimulates chronic GC reactions with high levels of atypical MBCs, which have been associated with autoimmune disease.^29,53–55^ To understand the dynamics of MBC differentiation during HIV infection, we investigated a dataset of bulk BCR sequences from lymph node biopsies of 3 HIV-1 infected donors.^29^ These donors were clinically estimated to have been infected 4 months to 3 years prior based on the strength and timing of HIV antibody tests (Fig. 4A, donor H1: 12–18mo, H2: 24–36mo, H3: 4–8mo; Dr. Susan Moir, personal communication). B cells were sorted into GC B cells (GCBC), unswitched MBC (UnMem), CD19^lo^ (classical MBC), and CD19^hi^ (atypical MBC). Each patient was sampled at one timepoint only, so type-specific clock rates were estimated externally. To obtain a GC clock rate estimate, we used previously identified measurably evolving B cell lineages from an HIV-1 infected donor sampled between 6–144 weeks after HIV-1 infection.^49,56^ We estimated the clock rate of each lineage using root-to-tip regression; i.e., slope of the linear regression of sample time vs genetic divergence from the MRCA.^35^ We used the mean rate of these 118 lineages (1×10^−3^ mut/site/week) as the mean of the normal prior clock rate for GC B cells in TyCHE and the fixed mean clock rate for SC and UCLD models (Fig. 4B). When using TyCHE, the clock rate of MBCs was *a priori* modeled by a normal distribution with mean 10^−6^ and standard deviation 0.001.

We first tested whether models correctly predicted GC B cells as the root cell type of each lineage. For each patient, we downsampled lineages to have equal numbers of GC B cells and MBCs. To make this test non-trivial, we allowed reversible switches from MBCs back to GCs. Of the 95 lineages tested, maximum parsimony trait reconstruction on genetic distance trees incorrectly placed MBCs at the root for 57 lineages (Fig. 4C–D). SC and UCLD models predicted MBC as the root cell type in 40 and 42 lineages, respectively. By contrast, TyCHE’s EO model correctly predicted the root as GC for 93 lineages (97.9%, Fig. 4D, Supplemental Fig. 7). We next tested whether models correctly inferred that GCs tended to produce MBCs, rather than the reverse.^57^ In all patients, TyCHE estimated a significantly higher transition rate from GC to MBC compared to the reverse, while in SC and UCLD models this was only observed in one patient (Fig. 4E). These results validate TyCHE’s ability to correctly infer B cell differentiation patterns in primary, chronic GC reactions.

We next used TyCHE to reconstruct the timing and MBC differentiation patterns from these GC reactions. First, we used TyCHE’s EO model with irreversible switches from the GC and fixed GC/MBC clock rates to estimate the root date of each lineage, representing the initiation date of the GC reaction. Strikingly, median estimated tree heights were either during or shortly after the clinically estimated time of infection for all three donors (Fig. 4F). We next estimated the time of differentiation for each MBC subset. We filtered to clones that contained all four B cells subsets and at least 20 distinct BCRs, then downsampled each to contain at most 100 sequences while representing each group as evenly as possible with no more than 50 cells from any subset. Similarly to Fig. 3F, we used a 4 state IS GC-irreversible model and calculated the date of each MBC’s most recent GC ancestor. We found that unswitched MBCs usually exited the GC earliest, followed by CD19^hi^ MBCs and finally CD19^lo^ MBCs, consistent with prior results that these atypical (CD19^hi^) MBCs exit the GC early and accumulate in lymph nodes (Fig. 4G). These two results (Fig. 4F–G) demonstrate that TyCHE can make clinically plausible predictions about the initiation dates of GC reactions as well as realistic differentiation patterns of MBCs.

### Recall GC reactions following repeated human influenza vaccination

**Figure 5:**
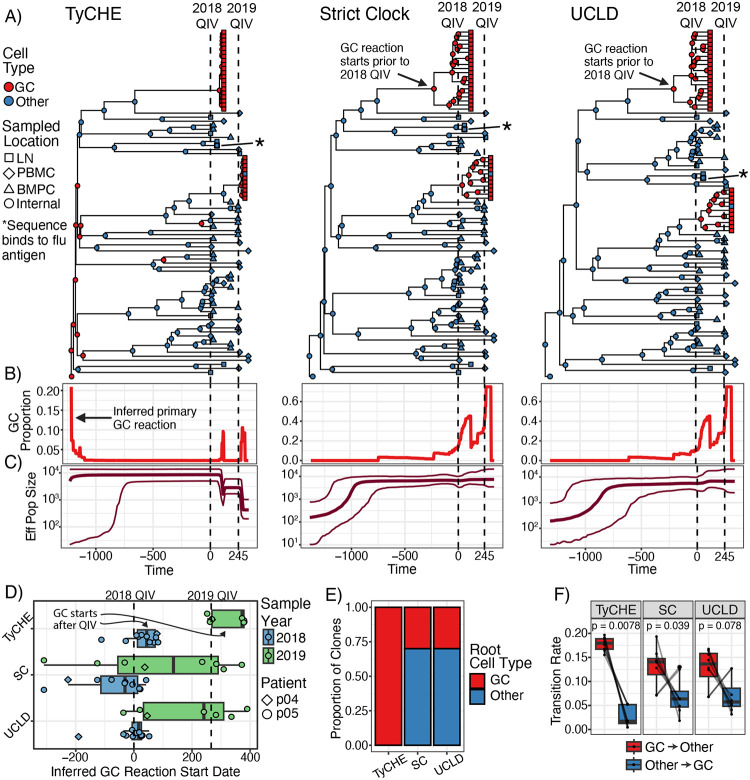
Time-resolved analysis of recall GC reactions following repeated influenza vaccination. Donors were immunized with 2018/2019 QIV at week 0 and re-immunized with 2019/2020 QIV at week 35 (P04) or 38 (P05). Samples were taken from PBMCs, lymph node (LN), and bone marrow plasma cells (BMPCs). **A)** Time trees of the largest lineage from P04. Dashed lines show dates of vaccinations. **B)** Proportion of branches predicted to be GC B cells. **C)** Bayesian skyline plots showing changes in effective population size. **D)** Inferred GC reaction start dates. Each dot represents a GC reaction. Boxplots and dots are colored by the flu season in which the GC B cells were sampled. Dotted lines show the dates of 2018 and 2019 QIVs for P05. **E)** Reconstructed cell type at the root for all lineages. **F)** Estimated transition rates from GC to other B cells and vice-versa. P values were calculated using the Wilcoxon signed-rank test.

In contrast to HIV infection that generates chronic GC reactions, influenza vaccination in adults elicits a memory response in which MBCs can form recall GCs.^30,52,56,58^ These lineages experience bursts of evolution during recall GCs preceded and followed by years of little evolution as MBCs. We used TyCHE to investigate a published dataset of single-cell BCR sequences from longitudinal blood, lymph node, and bone marrow samples obtained from two human adults following seasonal influenza vaccination over the 2018/2019 and 2019/2020 flu seasons (Fig. 5).^30^ Among these data, we identified 8 measurably evolving lineages that contained GC B cells sampled after each vaccination, and were experimentally confirmed to bind to influenza antigens using monoclonal antibodies. We used a clock rate of 4.9×10^−3^ mut/site/week for GC B cells in this analysis, which was the median slope from root-to-tip regressions of previously-identified measurably evolving lineages from one of these donors (P05) sampled for 60 days after the 2018/2019 flu vaccine.^56^

We tested whether TyCHE could infer the periodicity of recall GC reactions using these BCR sequences. TyCHE correctly predicted bursts of GC B cells following vaccination with long periods of MBCs in between (Fig. 5A–C, Supplemental Fig. 8–10). We defined a GC reaction as a contiguous set of internal nodes predicted to be GC B cells leading to sampled GC B cells. We estimated the date that each GC reaction began as the date of its earliest GC-inferred internal node. These GCs were presumably induced by the vaccine and should have begun only after vaccination (Fig. 5A). TyCHE correctly inferred that 80% of recall GCs began at or after the date of each season’s vaccination (Fig. 5D). By contrast, SC and UCLD models predicted that only 33% and 55%, respectively, of recall GC reactions began after each season’s vaccination, which is biologically implausible. Even less plausibly, SC and UCLD models predicted that, respectively, 38% and 14% of GCs sampled after the 2019/2020 vaccination began prior to the 2018/2019 vaccination (Fig. 5D, green boxplots). Strikingly, only TyCHE inferred the unsampled primary GC reaction at the root of each lineage (Fig. 5B, E). Using a Bayesian skyline plot of the largest clone from P04, only TyCHE inferred sharp decreases in effective population size during recall GCs, consistent with strong selection and clonal expansion (Fig. 5C). TyCHE also correctly predicted significantly higher transition rates from GC to MBCs (Fig. 5E). These results demonstrate that TyCHE can accurately reconstruct cycles of B cell evolution following repeated vaccination.

### Evolution of a hypermutating glioma tumor lineage

**Figure 6:**
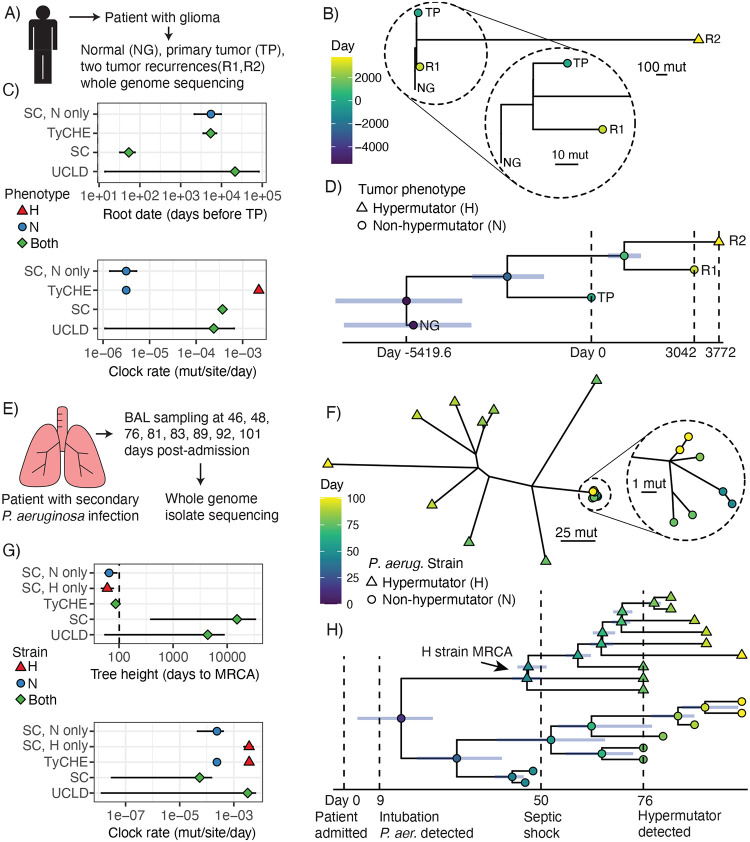
Time-resolved analysis of hypermutating glioma tumor lineage (A-D) and *Pseudomonas aeruginosa* infection (E-H). **A)** Tumor genome samples from a patient with glioma. **B)** Genetic distance tree of hypermutator (R2) and non-hypermutator (TP, R1) tumors rooted at NG. Non-hypermutator branches zoomed in for detail. This panel shares color/shape scales with panel D. **C)** Root date (above) and clock rate (below) estimates from each model, both in log_10_ scale. **D)** Time-resolved tumor phylogeny from TyCHE, tip color and shape shares scales with panel B. Blue bars show 95% highest posterior density (HPD) interval of node height. **E)** Bronchial alveolar lavage (BAL) samples from a patient with COVID-19 associated pneumonia and secondary *P. aeruginosa* infection. **F)** Unrooted genetic distance tree of hypermutator (H) and non-hypermutator (N) isolates. N clade zoomed in for detail. This panel shares color/shape scales with panel H. **G)** Tree height (above) and clock rate (below) estimates from each model, both in log_10_ scale. Tree height of ~101 days (dashed line) is expected as the patient showed no signs of lung infection before hospitalization. **H)** Time-resolved phylogeny of H and N isolates, shares color/shape scales with panel F. Blue bars show 95% HPD interval of node height. MRCA of all isolates estimated to be at/shortly after intubation. MRCA of H estimated shortly before the septic shock, and H lineage evolved in a ladder-like manner shortly afterwards.

Tumors frequently accumulate mutations over time, and phylogenetic analysis of these mutations can reveal significant insights into the response of tumors to treatment.^7^ Some tumors develop hypermutator phenotypes, which frequently occur in gliomas during temozolomide treatment. We used TyCHE to resolve the evolution of a mixed hypermutator (H)/non-hypermutator (N) glioma lineage in one patient (4F0A) profiled in the GLASS consortium (Fig. 6A).^31^ Samples included normal genome (NG), primary tumor (TP, day 0), recurrence 1 (R1, day 3042) and recurrence 2 (R2, day 3772). R2 was classified as a hypermutator based on mutation levels (Fig. 6B). We estimated the clock rate of N cells using the SC model on NG, TP, and R1, as well as the clock rate of H using root-to-tip regression of R1, R2, and NG. Using the SC model on only N sequences, we estimated the root date of the lineage at ~15.1 years before TP, consistent with slow-growing low-grade gliomas (Fig. 6C). Using all sequences, TyCHE estimated a similar root date of 14.8 years prior to TP (Fig. 6C–D). By contrast, the SC on all sequences estimated an implausibly recent root date of 53.5 days prior to TP, likely due to an inflated clock rate estimate, while the UCLD model predicted an implausibly early root date of 58.9 years before TP (patient age was 35 years at TP; Fig. 6C). These results demonstrate that TyCHE can reconstruct the temporal dynamics of complex, heterogeneous tumor populations with hypermutating subclonal populations.

### Evolution of a hypermutating bacterial lung infection

*P. aeruginosa* can cause deadly bacterial lung infections, which often begin with a single bacterium that rapidly adapts to the lung environment.^21^ Mutations in DNA repair enzymes produce hypermutator lineages that evolve far more rapidly than wild-type strains.^22,59^ We used TyCHE to reconstruct the evolution of a *P. aeruginosa* hypermutator lineage in an acute, fatal lung infection.^22^ Whole genome sequencing was previously performed on isolates obtained from bronchial alveolar lavage samples of a patient admitted for COVID-19 related pneumonia with no indication of prior *Pseudomonas* infection (Fig. 6E). *P. aeruginosa* was first detected on day 9, and on day 76 a hypermutator (H) strain was detected with genetic diversity far exceeding that of non-hypermutator (N) isolates (Fig. 6F). Using the SC model on H and N strains separately, we estimated similar MRCA dates of 40.3 and 35.4 days post-admission, respectively, but a much higher clock rate for H (Fig. 6G). Using these clock rate estimates for H and N strains, TyCHE estimated an MRCA date of all sequences at 14.4 days post-admission (95% HPD interval: 3.4, 23.9, Fig. 6H), overlapping with the patient’s first positive *P. aeruginosa* sputum sample at intubation (day 9). By contrast, SC and UCLD clock models estimated implausibly early root dates of 41.4 years and 11.7 years before admission, respectively (Fig. 6G). Using TyCHE, we estimated the MRCA of the H strain at day 46.5, shortly before the patient experienced septic shock on day 50. The H strain also exhibited a ladder-like branching pattern, consistent with rapid evolution, roughly corresponding to a two-week period of intense antibiotic treatment following septic shock (Fig. 6H).^22^ These results are consistent with the H strain contributing to septic shock and evolving resistance to antibiotics in the patient. Supporting this, peripheral blood samples taken at days 76, 78, and 79 contained only H isolates, and H isolates were resistant to more diverse antibiotics than N isolates.^22^ These results confirm that TyCHE can infer clinically plausible time-resolved phylogenies in hypermutating bacterial populations.

## Discussion

Inferring time-resolved phylogenies of cellular lineages could uncover new discoveries about how cell proliferation, differentiation, and migration shape responses to perturbations like vaccines and drug treatments. Unfortunately, mutation rates often vary significantly by cell type, biasing existing models. Here, we introduce TyCHE, which uses type-linked clock models to estimate accurate time trees for heterogeneously evolving populations. Using simulations, we show TyCHE estimates more accurate tree topologies, node dates, and ancestral cell types than existing methods. Further, we show how TyCHE can use BCR sequences to reconstruct both primary GC reactions in HIV infection and recall GC reactions following influenza vaccination. When applied to sequences from hypermutating tumor lineages and bacteria, TyCHE can infer clinically plausible and useful time trees. By contrast, existing SC and UCLD models produced implausible results. Further, we introduce SimBLE, which simulates both BCRs evolving during GC reactions and neutral heterogeneous evolution, both to benchmark these new models and perform *in silico* evolution experiments.

While most phylogenetic models that include clock rate heterogeneity allow rates to vary independently of cell type,^27,33^ some prior studies have linked evolutionary rate with discrete traits. One study introduced a likelihood-based method to detect shifts in trait-dependent evolution.^60^ More recently, the package SDevo linked tumor cell birth rates in a birth-death model to discrete states, specifically the position of cancer cells along the edges of a tumor.^61^ TyCHE differs from SDevo’s approach by allowing clock rates (rather than growth rates) to vary by cell type, introducing the EO approximation, and utilizing a simpler CTMC model. One limitation of TyCHE is that prior information about clock rates is sometimes needed, but this can be obtained using strict clock models or root-to-tip regressions applied to pure cell populations, as was done in this study. Because of this two-step process, similar to empirical Bayes methods, 95% highest posterior density intervals should always be interpreted as conditioned on the provided cell-type clock rates, especially if estimated from the same data. The examples tested in this study used cell types with rates differing by orders of magnitude. SC and UCLD models may be more competitive when analyzing cell types with more similar rates. Indeed, some recent studies have generated time trees based on somatic mutations, but did not link mutation rate to cell type.^62,63^ While in this study we used naturally-occurring somatic mutations, TyCHE could be applied to dynamically mutating lineage recorders if combined with an appropriate sequence substitution model (e.g.^64–66^).

We introduce TyCHE and SimBLE for accurately reconstructing and simulating heterogeneously evolving populations, respectively. Because type-linked mutational heterogeneity is a very general phenomenon, we believe the applications explored in this study are a small fraction of potential applications for these programs.

## Methods

TyCHE (Type-linked Clocks for Heterogenous Evolution) introduces a framework for time-resolved Bayesian phylogenetic inference on populations that evolve at different rates. TyCHE is implemented as a new BEAST2^28^ package and validated with SimBLE (Simulator of B cell Lineage Evolution). As with other BEAST2 packages, the type-linked models in TyCHE are specified using XML files. For ease of use, especially on multiple lineages in concert, we developed a system of functions within the R package Dowser^32^ that allows users to specify general XML templates applied to multiple lineages at once. A vignette showing how TyCHE can be used on both B cells and non-B cells is available at https://dowser.readthedocs.io.

### Type-linked clock model

The observed data comprise discrete states (e.g. cell types) $X=\left(X_{1},\ldots ,X_{N}\right)$ and aligned nucleotide sequences $Y=\left(Y_{1},\ldots ,Y_{N}\right)$ at the N tips of the phylogenetic tree F. For each tip, we observe a state $X_{i}\in S_{X}$ and a sequence $Y_{i}\in S_{Y}$, where $S_{x}$ is the set of all states and $S_{y}$ is the set of all potential nucleotide sequences of common length L. Neither states nor sequences are observed at the internal nodes of the phylogeny or the root; i.e. $X_{\text{root}},Y_{\text{root}},\left(X_{N+1},\ldots ,X_{2N-2}\right),\left(Y_{N+1},\ldots ,Y_{2N-2}\right)$ are unknown.

We adapt a Bayesian discrete ancestral state reconstruction method^36^ to model the state changes X(t) along the branches of the phylogeny F via continuous time Markov chains (CTMCs). CTMCs are stochastic processes in continuous time that emit discrete outcomes and are memoryless; i.e., ∀i,j∈S,∀s,t≥0,P(X(s+t)=j∣X(s)=i, {X(u):0≤u≤s})=P(X(s+t)=j∣X(S)=i). A two-state CTMC is fully specified by the instantaneous rate matrix $Q=\left(\begin{array}{cc} -\alpha & \alpha \\ \beta & -\beta \end{array}\right)$, which necessarily contains non-negative off-diagonal entries and rows that sum to 0. Solving the Chapman-Kolmogorov equation results in the finite-time state transition matrix $P(t)=e^{Qt}=\left\{{\text{P}}_{ij}(t)\right\}$, where ${\text{P}}_{ij}(t)=\text{P}(X(t)=j|X(0)=i)$. The sequences Y are similarly modeled with a CTMC which assumes each site mutates independently according to the instantaneous rate matrix $Q_{Y}$, resulting in the joint TyCHE Bayesian hierarchical model (BHM):

(1) $$\text{P}\left(F,Q,Q_{Y}|X,Y\right)\propto \text{P}\left(X,Y|F,Q,Q_{Y}\right)\text{P}\left(F,Q,Q_{Y}\right)=P\left(Y|X,F,Q_{Y}\right)P\left(Q_{Y}\right)\text{P}(X|F,Q)P(Q)P(F).$$

The phylogeny prior, P(F), can be chosen to be any valid tree prior. In this work, we use a Bayesian skyline tree prior^67^ in all analyses and validations unless otherwise specified.

The state instantaneous rate matrix Q can be decomposed into the product of the type-switch clock rate $\mu_{X}$, and a normalized instantaneous rate matrix $Q^{*}=\left(\begin{array}{cc} -\alpha^{*} & \alpha^{*}\\ \beta^{*} & -\beta^{*} \end{array}\right)$. The hyperparameter $\alpha^{*}=r_{AB}f_{A}$, where $r_{AB}$ is the relative rate of transition from state A to state B and $f_{A}$ is the relative frequency of state A. The hyperparameter $\beta^{*}=r_{BA}f_{B}$ is defined analogously. Therefore, the prior distribution of the state instantaneous rate matrix Q can be written $P(Q)=P\left(\mu_{X}\right)P\left(r_{AB}\right)P\left(f_{A}\right)P\left(r_{BA}\right)P\left(f_{B}\right)$. The relative frequencies of the states in our analyses are likely changing over time. As such, we wish to minimize the impact of the relative frequencies in the model, and set $f_{A}=f_{B}=0.5$ to avoid biasing $Q^{*}$.

The typed-tree likelihood, P(X∣F,Q), represents the likelihood of the internal node states given the observed states at the tips, the phylogeny F, and the state instantaneous rate matrix $Q=\mu_{X}*Q^{*}$. It is assumed that the sequence CTMC depends on the internal node states, but the state CTMC is conditionally independent of the sequences given the phylogeny F. The typed-tree likelihood can be decomposed into the product of the state transition probabilities along each branch i of $F,\text{P}(X|F,Q)=\prod_{i}P\left(X_{\text{child}}|X_{\text{parent}},t_{i},Q\right)=\prod_{i}P\left(X_{\text{child}}|X_{\text{parent}},t_{i}*\mu_{X},Q^{*}\right)$, where $X_{\text{child}}$ is the state of the child node on branch $i,X_{\text{parent}}$ is the state of the parent node on branch i, and $t_{i}$ is the length in time of branch $i;\mu_{X}$ is the type-switch clock rate such that the product $t_{i}*\mu_{X}$ represents the distance between parent and child types, and $Q^{*}$ is the normalized instantaneous rate matrix. This represents the joint likelihood of state transitions given the states at each internal node, and differs from other approaches^36^ which assume node states do not affect clock rates and therefore sample states from the marginal posterior probabilities at each node.

The nucleotide substitution instantaneous rate matrix $Q_{Y}$ is specified by the chosen nucleotide substitution model. Unless otherwise noted, the HKY substitution model^68^ was used with equilibrium nucleotide frequencies fixed to their empirical frequencies, and a prior was placed on the transition/transversion rate ratio κ, so $P\left(Q_{Y}\right)=P(\kappa )$.

The tree likelihood $P\left(Y|X,F,Q_{Y}\right)$ is calculated as a standard BEAST2 tree likelihood.^28^ Briefly, a clock model is used to convert the branches of the tree F into a genetic distance tree, and the likelihood of that tree is calculated using Felsenstein’s pruning algorithm.^34^ As in a relaxed clock model^27^ or random local clock model,^33^ in TyCHE each branch receives an individual rate of evolution, or branch rate, which is defined as the rate of genetic mutation for the sequences Y along a given branch of the phylogeny F. Unlike in previous models, in TyCHE the branch rate is linked to the state of the parent and child nodes.

For ease of notation, let the binary state space $S_{X}=\{A,B\}$, and denote the state-specific mutation rates as $\mu_{A}$ and $\mu_{B}$. We present two distinct branch rate models in TyCHE: the type-linked evolutionary rate expected occupancy time (EO) model and the type-linked evolutionary rate instant switch (IS) model.

The principal model in TyCHE, the EO model, is implemented for a two-state case and determines the expected amount of time spent in each state along a branch as a function of the parent and child node states and the instantaneous rate matrix Q. Then the overall branch rate is

(2) $$\mu_{\text{branch}}=\frac{\mu_{A}E\left[O_{A}|\ldots \right]+\mu_{B}E\left[O_{B}|\ldots \right]}{t_{\text{branch}}},$$

where $E\left[O_{A}|\ldots \right]$ is the expected occupancy time in state A given the parent state, child state, and instantaneous rate matrix $Q,E\left[O_{B}|\ldots \right]$ is analogous for state B, and $t_{\text{branch}}$ is the length of the branch in time. Note that $t=E\left[O_{A}|\ldots \right]+E\left[O_{B}|\ldots \right]$, so this is a weighted average of the state-specific mutation rates. The derivation of the expected occupancy times is given in the following section.

TyCHE’s IS model sets $\mu_{\text{branch}}=\mu_{\text{child}}$, where $\mu_{\text{child}}$ is the mutation rate of the child state. Essentially, this model assumes that the entirety of the branch is spent in the state of the child node. For both the EO and IS models, we provide the option for users to either estimate the state-specific mutation rates or provide fixed values.

### Calculating the expected occupancy time

In TyCHE’s expected occupancy time model, the expected occupancy time in each state along each branch of the phylogeny F is determined by the instantaneous rate matrix $Q=\left(\begin{array}{cc} -\alpha & \alpha \\ \beta & -\beta \end{array}\right)$, the time length of the branch, and the parent and child states. For ease of notation, let the binary state space $S_{X}=\{A,B\}$, and set the time such that $t_{\text{parent}}=0$ and $t_{\text{child}}=t$. Then the state transition matrix is along the branch is $P(t)=e^{Qt}=\left(\begin{array}{ll} {\text{P}}_{AA}(t) & P_{AB}(t)\\ P_{BA}(t) & P_{BB}(t) \end{array}\right)$, where ${\text{P}}_{ij}(t)=\text{P}(X(t)=j|X(0)=i)\forall i,j\in S_{x}$.

As an example, let X(0)=A,X(t)=B, i.e., the parent state is A and the child state is B. Per ^69^ the occupancy time in state A can be written as

(3) $$O_{A}=\int_{0}^{t}\text{I}\left(X\left(s\right)=A\right)ds,$$

where I(⋅) is the indicator function. Then the expected value of the occupancy time in state A given the parent and child states is

(4) $$\text{E}\left[O_{A}|X(0)\right.=A,X(t)=B]=\text{E}\left[\int_{0}^{t}\text{I}(X(s)=A)ds|X(0)=A,X(t)=B\right]\;=\int_{0}^{t}\text{E}[\text{I}(X(s)=A)|X(0)=A,X(t)=B]ds\;=\int_{0}^{t}\text{P}\left(X\left(s\right)=A|X\left(0\right)=A,X\left(t\right)=B\right)ds.$$

In these expressions, the order of the expectation and integration are interchanged by Fubini’s theorem, and the expectation of the indicator function is the simple probability. Consider now the expression P(X(s)=A∣X(0)=A,X(t)=B). By a conditioning argument,

(5) $$\text{P}\left(X\left(s\right)=A|X\left(0\right)=A,X\left(t\right)=B\right)=\frac{\text{P}\left(X\left(s\right)=A,X\left(t\right)=B|X\left(0\right)=A\right)}{\text{P}\left(X\left(t\right)=B|X\left(0\right)=A\right)},$$

and $\text{P}(X(t)=B|X(0)=A)={\text{P}}_{\text{AB}}(\text{t})$. By a similar conditioning argument,

(6) $$\text{P}\left(X\left(s\right)=A,X\left(t\right)=B|X\left(0\right)=A\right)\;=\text{P}\left(X\left(t\right)=B|X\left(s\right)=A,X\left(0\right)=A\right)*\text{P}\left(\text{X}\left(\text{s}\right)=\text{A}|\text{X}\left(0\right)=\text{A}\right)\;=P\left(X\left(t\right)=B|X\left(s\right)=A\right)*\text{P}\left(\text{X}\left(\text{s}\right)=\text{A}|\text{X}\left(0\right)=\text{A}\right)\;={\text{P}}_{AB}\left(t-s\right){\text{P}}_{AA}\left(s\right).$$

Therefore,

(7) $$\text{E}\left[O_{A}|X(0)=A,X(t)=B\right]=\int_{0}^{t}\frac{{\text{P}}_{AB}(t-s){\text{P}}_{AA}(s)}{{\text{P}}_{\text{AB}}(\text{t})}.$$

To solve this expression, it is necessary to determine the forms of each of the functions in the state transition matrix $P(t)=e^{Qt}=\left(\begin{array}{ll} {\text{P}}_{AA}(t) & P_{AB}(t)\\ P_{BA}(t) & P_{BB}(t) \end{array}\right)$. Consider the eigen-decomposition $Q=VDV^{-1}$. The eigenvalues of Q are $\lambda_{1}=0,\lambda_{2}=-(\alpha +\beta )$, and the eigenvectors of Q are taken to be v1=11 and v2=-αβ, so $V=\left(\begin{array}{cc} 1 & -\alpha \\ 1 & \beta \end{array}\right)$ and $D=\left(\begin{array}{cc} 0 & 0\\ 0 & -(\alpha +\beta ) \end{array}\right)$. Then,

(8) $${\text{e}}^{Qt}=e^{\left(VDV^{-1}\right)t}=\sum_{k=0}^{\infty }\frac{t^{k}{\left(VDV^{-1}\right)}^{k}}{k!}\;=\sum_{k=0}^{\infty }\frac{t^{k}\left(VDV^{-1}VDV^{-1}V\ldots V^{-1}VDV^{-1}\right)}{k!}\;=\sum_{k=0}^{\infty }\frac{t^{k}\left(VDIDI\ldots IDV^{-1}\right)}{k!}=\sum_{k=0}^{\infty }\frac{t^{k}VD^{k}V^{-1}}{k!}=V\left(\sum_{k=0}^{\infty }\frac{t^{k}D^{k}}{k!}\right)V^{-1}=Ve^{Dt}V^{-1}.$$

As D is diagonal,

(9) $$e^{Dt}=\left(\begin{array}{cc} e^{0t} & 0\\ 0 & e^{-(\alpha +\beta )t} \end{array}\right)=\left(\begin{array}{cc} 1 & 0\\ 0 & e^{-(\alpha +\beta )t} \end{array}\right).$$

Thus,

(10) $$P\left(t\right)=e^{Qt}=Ve^{Dt}V^{-1}=\frac{1}{\alpha +\beta }\left(\begin{array}{cc} \beta +\alpha e^{-\left(\alpha +\beta \right)t} & \alpha \left(1-e^{-\left(\alpha +\beta \right)t}\right)\\ \beta \left(1-e^{-\left(\alpha +\beta \right)t}\right) & \alpha +\beta e^{-\left(\alpha +\beta \right)t} \end{array}\right).$$

Inserting these expressions into Equation (7), the expected occupancy time is

(11) $$\text{E}\left[O_{A}|X(0)=A,X(t)=B\right]=\int_{0}^{t}\frac{\left(\frac{\beta }{\alpha +\beta }+\frac{\alpha }{\alpha +\beta }e^{-(\alpha +\beta )s}\right)\times \left(\frac{\alpha }{\alpha +\beta }\right)\left(1-e^{-(\alpha +\beta )(t-s)}\right)}{\frac{\alpha }{\alpha +\beta }\left(1-e^{-(\alpha +\beta )t}\right)}ds.$$

Solving the integral analytically leads to the result

(12) $$\text{E}\left[O_{A}|X(0)=A,X(t)=B\right]=\frac{1}{\alpha +\beta }\left(\frac{\beta t-\alpha te^{-(\alpha +\beta )t}}{1-e^{-(\alpha +\beta )t}}+\frac{\alpha -\beta }{\alpha +\beta }\right).$$

While the occupancy time in state B can be solved for similarly, it is simpler to consider that, for a two state sample space $S_{X}$,

(13) $$\text{t}=\text{E}\left[O_{A}|X(0)=A,X(t)=B\right]+\text{E}\left[O_{B}|X(0)=A,X(t)=B\right],$$

and thus,

(14) $$\text{E}\left[O_{B}|X(0)=A,X(t)=B\right]=t-\frac{1}{\alpha +\beta }\left(\frac{\beta t-\alpha te^{-(\alpha +\beta )t}}{1-e^{-(\alpha +\beta )t}}+\frac{\alpha -\beta }{\alpha +\beta }\right).$$

Expressions for different combinations of parent states X(0) and child states X(t) can similarly be derived and are presented in Table 1.

### SimBLE: realistic germinal center reaction simulations

SimBLE is an open-source Python package that simulates type-linked evolution, either neutral evolution of random sequences, or BCR sequences from B cells in GC reactions using a realistic model of SHM, affinity maturation, and differentiation. SimBLE models essential processes including B cell competition for antigen in GCs and their differentiation into MBCs and plasma cells. Briefly, SimBLE picks a naïve cell with paired heavy and light chain and adds it to the *in silico* GC. If simulated under selection, at each generation the cells in the GC are evaluated for relative affinity and reproduce based on that affinity. Cells migrate out of GCs as MBCs or plasma cells at a user-specified rate, where typically they do not mutate and reproduce slowly with no selection.

To create a SimBLE naïve cell, a paired heavy and light chain are randomly selected from a previously curated dataset of naïve B cells.^37,38^ These cells were obtained from healthy donors and COVID-19 patients. Only cells transcriptionally identified as naïve B cells and containing unmutated heavy and light chain BCRs were included. The selected cell is added to the GC, where its relative fitness (affinity) is assessed based on similarity to a pair of target heavy and light chain BCR AA sequences, similar to the approach used in *bcr-phylo*^40^ for heavy chains. The target sequence is generated by mutating the CDRs of the germline sequence; by default, the target sequence has 5 single nucleotide AA substitutions in the heavy chain and 3 in the light chain. The number of target sites may be specified by the user. Single nucleotide AA substitutions are accessible by a single nucleotide substitution from the germline. The position of these substitutions in the target sequence (target mutations) are drawn randomly from within the CDRs which determine antigen binding. Target mutations were not allowed in the FWRs because those regions are usually conserved for antibody structure.

To replicate the targeting biases of SHM, mutations are added to BCRs according to the S5F model of SHM targeting and nucleotide substitution.^39^ When a child cell is created, n mutations are drawn for the heavy chain from a Poisson distribution with rate = (cell type’s relative mutation rate) * (expected heavy chain mutations per site) * (number of heavy chain sites). The same process determines mutations for the light chain. By default, GC B cells have a relative mutation rate of 1, and non-GC B cells have a relative mutation rate of 0, i.e., they do not mutate. Affinity of a cell’s BCR sequence is calculated based on mismatches from the target AA sequence, with different selection multipliers (weights) for each site. A site multiplier at the lower bound of 1 represents neutral impact of that site on binding ability. A multiplier greater than 1 represents increasing impact of that site on binding ability. Selection multipliers are chosen based on selection strength, m. Unless otherwise specified, m=2 throughout this work. CDR positions each have a selection multiplier randomly drawn from an exponential distribution such that the multiplier is always greater than or equal to 1 and 99.5% of multipliers are less than m. FWR positions have a selection multiplier randomly drawn from an exponential distribution such that the multiplier is always greater than or equal to 1, and 85% of multipliers are less than m. Target sites have a multiplier of m. By default, the distribution of site multipliers allows for most sites in the CDRs to evolve with near neutrality except for substitutions matching the target sequence, which are highly rewarded. The FWR distribution allows fewer sites to mutate with near neutrality than in the CDRs, while most sites will operate under purifying selection, reflecting that certain sites are more important to the structure and functionality of the FWR. The affinity score represents the product of the site multipliers for sites that match between the BCR and target AA sequence, and of the inverse site multipliers (1/m) at all sites that differ between the BCR AA and target AA sequence.

Let S be the set of positions that an amino acid sequence A has in common with the target sequence T and let D be the set of positions that are different, so that

(15) $$S=\left\{x:T_{x}=A_{x}\right\}\;D=\left\{x:T_{x}\neq A_{x}\right\},$$

where A is the amino acid sequence, $A_{x}$ is the xth position of A,T is the target AA sequence, and $T_{x}$ is the xth position of T.

Then the affinity score α of the amino acid sequence A of length n is:

(16) $$\alpha =\prod_{i=1}^{n}f\left(m_{i}\right),f\left(m_{i}\right)=\left\{\begin{array}{ll} m_{i}, & i\in S\\ \frac{1}{m_{i}}, & i\in D \end{array}\right.$$

The affinity score of a cell is the sum of the affinity scores of its heavy chain and light chain. If a cell has a premature stop codon in either chain, its affinity is set to zero. Biologically, the affinity score of a cell represents how well that cell’s BCR binds to the target antigen. To simulate competition and affinity-driven selection, SimBLE GCs have a fixed number of antigen particles available, which are distributed to cells with probability proportional to their relative affinity. Cells proliferate according to the number of antigen molecules bound, up to 10 child cells. Reproduction in the germinal center is modeled as sequential rounds of proliferative clonal bursting with transiently silenced SHM, followed by SHM of descendent cells.^42,43^ Algorithmically, this is equivalent to a cell having c direct children who each undergo one round of mutation. Thus, cells proliferate according to their amount of antigen bound by having c direct children, where c=min(10,numberofantigenmoleculesbound). As such, if a cell binds to no antigen, it dies within a generation.

Under neutral evolution, affinity is not calculated and antigen particles are distributed to cells randomly with equal probabilities rather than with probabilities weighted by affinity (Supplemental Fig. 2). To model differentiation of MBCs and plasma cells, for each generation we draw n, the number of cells to differentiate and exit the GC, from a Poisson distribution with rate specified by the user (set to 2 cells/generation in all analyses presented unless otherwise specified). Based on the current generation, $t_{gen}$ and generations per day parameter (default 2 generations per day), SimBLE determines what percentage of n should become MBCs, $x_{MBC}$, and what percentage should become plasma cells, $x_{PC}$, according to observed kinetics of differentiation.^41^ From observed patterns, SimBLE sets

(17) $$x_{MBC}=\left\{\begin{array}{cc} 0.99, & t<8\\ 0.99-0.1225t, & 8\leq t<16\\ 0.01, & 16\leq t<41\\ 0, & t\geq 41 \end{array}\right.$$

where $t=t_{\text{gen}}$ · generations per day, and represents the current day.

SimBLE then randomly selects $n_{MBC}=int\left(x_{MBC}\cdot n\right)$ cells from the GC population to differentiate into MBCs and selects $n_{PC}=n-n_{MBC}$ cells to differentiate into plasma cells from the GC population with probability proportional to their affinity. This matches observed stochasticity of MBC differentiation, particularly in regard to affinity, and high-affinity plasma cell differentiation.^70,71^ The newly-differentiated MBCs and plasma cells are removed from the germinal center and added to the population in the “other” compartment.

### Simulating evolution of other sequence types using SimBLE

For applications not involving GC reactions, SimBLE can simulate uniform neutral evolution of random sequences from heterogeneously evolving populations. In this case, a randomly generated nucleotide sequence of user-specified length is used as the starting sequence rather than an observed naïve BCR, mutations are introduced randomly rather than according to known hotspot motifs^39^, and antigen particles are distributed to cells randomly with equal probabilities rather than with probabilities weighted by affinity (Supplemental Fig. 2).

### Pipeline for standard TyCHE analysis

To facilitate using TyCHE under similar specifications on multiple lineages, we developed a template-based system for running TyCHE from within the R package Dowser v2.4.0. BEAST2 uses XML files to specify data, model structure, prior values, and MCMC settings. We developed customizable XML templates which can be applied to multiple lineages at once. Users process data within the standard Dowser pipeline and can then specify the XML template with the desired model specifications using the getTimeTrees or getTimeTreesIterate functions. The function getTimeTrees will run BEAST2 with a specified XML template on a collection of lineages for a specified number of MCMC steps. Expanding on this function, getTimeTreesIterate will run getTimeTrees under the specified settings, assess convergence of desired parameters (e.g. effective sample size of at least 200), and resume the MCMC chain until either all parameters have converged or the maximum number of iterations is met. For analyses that include germline sequences (e.g. B cells and tumors), the rootfreqs package is used to set the MRCA as identical to the germline sequence.^72^ To allow for fixed differences between the germline and observed sequences, the germline sequence is also included as a tip. The date of this germline tip is sampled via MCMC through a random walk. Within Dowser, tree objects are imported using treeio and plotted using ggtree packages.^73,74^

### Simulation-based validation

TyCHE was validated through simulation-based analyses using simulations of primary and recall GCs under selective evolution and uniform neutral evolution. In all simulations, 20 clones were simulated and a total of 96 cells were sampled from GC and non-GC compartments at four distinct timepoints. In primary GC reaction simulations, 24 B cells are sampled at 50, 100, 150, and 200 generations. To simulate recall GC reactions, we simulated a primary GC for 100 generations, sampling 12 cells from the GC and 12 cells from the other compartment at generations 50 and 100. After the primary GC reaction finished, we randomly selected an unsampled memory B cell to wait 1000 generations then re-enter a distinct GC and seed the recall reaction. Functionally, the age of this cell was increased by 1000 generations, and we ran a new SimBLE simulation with this cell as the progenitor, beginning at generation 1100. To represent a new perturbation, such as a new vaccine, the target sequence for the secondary GC reaction was a mutated version of the original GC reaction’s target sequence, with 5 additional single nucleotide AA substitutions in the heavy chain and 3 single nucleotide AA substitutions in the light chain. Other than the mutated target sequence, the secondary GC reaction is configured to use identical model parameters and settings to the first GC reaction, sampling 12 B cells from the GC and 12 cells from the other compartment at generations 1150 and 1200.

Three variants of TyCHE, the EO model with fixed and estimated clock rates as well as the IS model with estimated clock rates, were benchmarked against the existing SC and UCLD models using standard discrete trait reconstruction.^27,36^ The IS model with fixed clock rates performed poorly in initial simulations, so was not included. We sampled posterior distributions using MCMC with a chain length of 10^8^ iterations for primary GC simulations and 10^9^ iterations for recall GC simulations via the getTimeTreesIterate function from Dowser, with up to 9 additional chains of the same length or a maximum run time of one week for primary GC simulations and three weeks for recall GC simulations. While all models converged for primary GC simulations, in recall GC simulations 8 clones failed to converge for at least one model and were removed from comparisons for all models. In primary GC reaction simulations, all models assumed GC-irreversibility, i.e., GC B cells were allowed to differentiate into non-GC B cells, but not the reverse. Naturally, in recall GC reaction simulations all models assumed reversibility, i.e., GC B cells can differentiate into non-GC B cells and non-GC B cells can re-enter the GC.

For TyCHE models, the GC clock rate was initially estimated by the SC model applied to solely the GC B cells (only the primary GC reaction in the recall GC simulations). Then, the GC clock rate was either fixed or used this value with a strong prior. For the EO and IS models with estimated clock rates, the GC clock rate was modeled as a strong normal prior distribution with mean equal to the GC clock rate previously estimated only on GC B cells and standard deviation equal to 10^−2^ × previously estimated GC clock rate. The non-GC B cell clock rate was given a weaker normal prior distribution with mean 1 × 10^−6^ and standard deviation 1 × 10^−3^. For all models, we used a Bayesian Skyline tree prior with 5 intervals, the HKY substitution model,^68^ a lognormal prior on the transition/transversion rate ratio kappa with M=0.67 and S=0.2 (representing a mean ~2), and empirical nucleotide frequencies. The getTimeTreesIterate function in Dowser was used to sample the posterior distribution using MCMC with a chain length of 10^8^ steps and 2000 samples, with 200 labeled tree samples. If convergence was not reached, the MCMC chain was resumed for an additional 10^8^ steps up to 10 times. Convergence was evaluated with the criteria of all parameters operated on, except rate categories and internal node states, reaching an effective sample size of greater than 200 for the primary GC reaction simulations and greater than 100 for the GC recall simulations. A lower ESS threshold was used for recall GCs due to difficulty in reaching convergence compared to primary GC simulations. Clones that did not converge were eliminated for all comparisons. A total of 8 clones were removed due to lack of convergence in at least one model for uniform neutral GC recall simulations. All models converged for all other clones across simulation scenarios.

Estimated time trees were compared to the ground truth from SimBLE. Specifically, we assessed the tree height, cell type accuracy (percentage of correctly inferred cell types for each MRCA of all pairwise combinations of tips), and the Robinson-Foulds distance after collapsing branches with length less than 5 generations for the evaluation of tree topology. We also assessed tree length; i.e., sum of all branch lengths. Differentiation time was calculated using the height of each non-GC B cell’s most recent ancestral GC node, normalized by the full tree height. To understand the effects of biased sampling, primary GC reaction simulations were repeated with a 1:3 sampling of GC and non-GC B cells (Supplemental Fig. 5).

### Analysis of chronic GCs in HIV

Bulk BCR sequences from chronic GC reactions during HIV-1 infection were obtained from a prior study of three HIV-1 infected patients.^29^ Briefly, lymph node biopsies were obtained from donors H1, H2, and H3 (donors HIV1, 2, and 3 in original study, respectively), who were estimated to have been infected with HIV-1 for approximately 12–18mo, 24–36mo, and 4–8mo based on prior testing history and strength of anti-HIV antibody tests (Dr. Susan Moir, personal communication). Using fluorescence-activated cell sorting, B cells were sorted into GCBC, unswitched MBC, CD19^hi^ MBC, and CD19^lo^ MBC populations, which were sequenced separately using PCR-based amplification of BCR genes and Illumina MiSeq. Preprocessing, quality control, and identification of B cell clones were performed in ^29^.

Because sequences were collected from a single timepoint, it was not possible to estimate clock rates from these data. Instead, we estimated a clock rate using a prior longitudinal study of an HIV-1 infected patient over the first 3 years of infection.^49^ Measurably evolving lineages from this dataset, which accumulated a significantly higher SHM frequency over the sampled time period, were previously identified in ^56^. We estimated the clock rate of each of these lineages as the slope of its root-to-tip regression. We used the mean of these slopes (1 × 10^−3^ mut/site/week) as the mean clock rate for GCBCs in TyCHE models and for all cells in SC and UCLD models. For TyCHE models this value was set as the mean of a strong normal prior distribution with a sigma value of 10^−3^ × GC clock rate, effectively fixing it. The MBC clock rate used a weaker normal prior distribution with mean 1 × 10^−6^ and standard deviation 1 × 10^−3^. To prevent issues with non-identifiability of clock rate and tree height, for the SC model the clock rate was fixed to 1 × 10^−3^. For the UCLD, the mean clock rate was fixed at 1 × 10^−3^, but the standard deviation was estimated using MCMC. For all models we used a Bayesian Skyline tree prior with 5 intervals as well as a lognormal prior on the transition/transversion rate ratio kappa with M=0.67 and S=0.2. For each relative transition rate, we used a gamma distribution with shape α=0.1 and rate β=1.

The getTimeTreesIterate function in Dowser was used to sample the posterior distribution via MCMC with a chain length of 10^8^ steps and 2000 samples, with 200 labeled tree samples. Convergence was assessed by whether all parameters that were operated on, except rate categories and internal node types, reached an effective sample size of at least 200. If convergence was not reached, the MCMC chain was resumed for an additional 10^8^ steps up to 15 times. Clones that did not reach convergence in all models were discarded. For analyses in Figs. 4B–E, clones were downsampled to represent GC and non-GC B cells as evenly as possible, with a maximum of 25 cells in each population per clone. Clones with fewer than 20 distinct BCR sequences were discarded. For the analysis in Fig. 4F, only clones with all four B cell subtypes and at least 20 distinct BCR were included and were then downsampled to a maximum of 100 overall B cells and no more than 50 cells of any subtype.

### Analysis of repeated influenza vaccination

Single-cell RNA and BCR sequences were obtained from a prior study from three donors (P04, P05, P11) that received the 2018 and 2019 Flucelvax QIV from Sequirus.^30^ Because lymph node (LN) samples were not available from P11, only data from donors P04 and P05 were used in this study. No donors had received a flu vaccine in the preceding three years. All donors received the 2018 QIV at week 0. P04 received the 2019 QIV at week 35 and P05 received the 2019 QIV at week 38. Samples were obtained from peripheral blood mononuclear cells (PBMCs), lymph node fine needle aspirations (FNAs), and bone marrow plasma cells (BMPCs). Samples from P04 were obtained from sorted plasmablasts (PBs) at weeks 1 and 36, enriched IgD^lo^ B cells at weeks 0, 2, 17, 35, 37, 48, whole FNA at weeks 0, 1, 2, 17, 35, 37, 44, 48, and enriched BMPCs at weeks 0, 4, 26, 35. For P05, samples were obtained from whole PBMCs at weeks 13 and 17, enriched IgD^lo^ B cells at weeks 13, 17, 26, 38, 39, 40, 42, 47, 51, whole FNA at weeks 13, 26, 38, 39, 42, 47, 55, and enriched BMPCs at weeks 0, 4, 13, 26, 38, 42, 51. Samples were sequenced using the 10X Genomics Single Cell 5’ RNAseq with BCR amplification. Preprocessing, quality control, and identification of B cell clones and subtypes were performed in ^30^. To determine whether particular clones reacted to influenza, monoclonal antibodies (mAbs) were previously derived from selected clones and tested for binding affinity to influenza antigens. Clones were determined to be measurably evolving if they accumulated significantly higher SHM frequency over the sampled time period as determined by the correlationTest function in Dowser.^56^ We analyzed all clones containing an influenza-binding mAb sequence, that had GC B cells sampled after both vaccinations, and were measurably evolving. This resulted in 1 clone from P04 and 7 from P05. Clones with greater than 100 total sequences were downsampled to 100 sequences while ensuring maximum balance between sampled B cell subtypes and tissues.

For type-linked analysis with TyCHE (EO with estimated rates), the GC clock rate used a normal prior distribution with mean equal to 4.9 × 10^−3^ mut/site/week and standard deviation equal to 10^−2^ × the mean. The clock rate for the other B cell subtypes used a normal prior distribution with mean equal to 1 × 10^−6^ and standard deviation equal to 1 × 10^−3^. The GC clock rate was estimated as the median slope of the root-to-tip regression for previously identified measurably evolving lineages from P05 sampled after the 2018 QIV vaccination (days 0–60).^52,56^ For all models, we used a Bayesian Skyline tree prior with 5 intervals and a lognormal prior on the transition/transversion rate ratio kappa with an M=0.67 and S=0.2. The priors on all cell type relative transition rates were set to gamma distributions with shape = 0.1 and scale = 1.0. To help with convergence, the maximum root date of the trees was set to four years prior to the 2018 QIV vaccination, as neither patient had received a flu vaccine in the preceding three years. While the true root date of these lineages was potentially earlier, our ability to resolve this date was low due to the arbitrarily low clock rate of MBCs. Thus, estimated root dates may be more recent than their true values. The getTimeTreesIterate function in Dowser was used to sample the posterior distribution via MCMC with a chain length of 10^9^ steps and 2000 samples as well as 200 state-labeled tree samples. If convergence was not reached, the MCMC chain was resumed for an additional 10^9^ iterations up to 10 times. Sampled posteriors were determined to have converged if all parameters that were operated on, excepting rate categories and internal node types, reached an effective sample size of at least 200.

When determining the proportion of branches predicted to be GC B cells over time for TyCHE (Fig. 5B, Supplemental Fig. 8B–10B), we calculated the expected occupancy of each branch in the GC state using the posterior maximum clade credibility tree, the posterior estimates of the relative transition rates among GC to non-GC cells, and the calculations for the expected occupancy time shown previously. For any ambiguous nodes on a branch, the occupancy time in the GC state is calculated for each of the potential states of the ambiguous node and averaged. For non-TyCHE models, we assumed that all branches are in the same state as the child node.

### Analysis of hypermutating glioma tumors

Sequences of glioma tumors were obtained from the GLASS Consortium.^31,75^ Patient 4F0A was selected due to multiple non-hypermutating samples and presence of a hypermutator phenotype in one sample. Sequences from this patient included normal genome (NG), primary tumor (TP, day 0), first recurrence (R1, non-hypermutator based on mutation level, day 3042), and second recurrence (R2, hypermutator, day 3722). Clinical and sequencing records for patient case barcoded GLSS-HF-4F0A were downloaded from the Synapse Repository (syn17038081). Variant calls from the 2022 updated consensus variant call format (VCF) files originally published in ^31^ were additionally filtered for likely germline variants by comparing them to recent releases of dbSNP (2025-03-12) and gnomAD (2024-10-11). Multiple sequence alignments were generated by first filtering VCF files to variants of size 1. Then, for any site mutated in at least one of the sequenced samples, a concatenated alignment was generated across mutant sites, using the paired-normal reference allele in instances where a mutation was not observed in a particular sample.

Genetic distance tree topologies, branch lengths, model parameters were estimated using the *dnaml* program of PHYLIP v3.695.^76^ We estimated the mean clock rate of non-hypermutators (N) using a strict clock model applied to only NG, TP, and R1. Due to the limited number of sequences, we estimated the clock rate of the hypermutator (H) using a root-to-tip regression from the genetic distance tree of NG, R1 and R2. For cell type specific clock rates in TyCHE, we used a normal prior with a mean equal to the estimated clock rates of H and N strains and sigma values equal to their respective mean × 10^−3^, effectively fixing these rates. Switches were assumed to be irreversible from N to H. Due to low sequence count, all models used a coalescent constant population size tree prior. A prior on kappa was set using a lognormal distribution with M=1.25 and S=0.5. Empirical nucleotide frequencies were used. The getTimeTreesIterate function in Dowser was used to sample the posterior distribution via MCMC with a chain length of 10^9^ iterations and 2000 samples and 200 state-labeled tree samples. Convergence was assessed by whether each parameter operated on, except rate categories and cell type states, reached an effective sample size of at least 200. If convergence was not reached, the MCMC chain was resumed for an additional 10^9^ iterations up to 20 times.

### Analysis of P. aeruginosa lung infection

*P. aeruginosa* sequence alignments were obtained from a prior study.^22^ Isolates were obtained from bronchial alveolar lavage samples from a patient admitted for COVID-19 related pneumonia with no sign of *P. aeruginosa* lung infection at 46, 48, 76, 81, 83, 89, 92, and 101 days after admission. Isolates were sequenced using Illumina NextSeq. Sequence processing, assembly, SNP identification, and sequence alignment were performed in ^22^. For computational expediency, only sites with SNPs were included in the alignment. Genetic distance tree topology, branch lengths, and HKY model parameters were estimated using maximum likelihood as implemented in the *phangorn* R package.^77^ Clock rates and tree heights of the non-hypermutator (N) and hypermutator (H) strains were estimated using SC models on those sequences separately. For TyCHE models, we used these mean clock rates as the mean of the normal prior for clock rates of N and H respectively, with a sigma value of 10^−3^ × the prior mean. This effectively fixed the clock rate for each type. Switches from N to H were assumed to be irreversible. In all models, the HKY substitution model was used as well as a Bayesian Skyline tree prior with 5 intervals. A prior on the transition/transversion rate ratio was set using a lognormal distribution with M=1.25 and S=0.5. The getTimeTreesIterate function in Dowser was used to sample the posterior distribution via MCMC with a chain length of 10^9^ iterations and 2000 samples. Convergence was assessed by whether all parameters that were operated on, except rate categories and internal node types, reached an effective sample size of at least 200. If convergence was not reached, the MCMC chain was resumed for an additional 10^9^ iterations up to 10 times.

## Supplementary Material

Supplement 1

## Figures and Tables

**Table 1:** Expected occupancy times given starting and ending states along a branch of length t.

X(0)	X(t)	Expected Occupancy Time
A	B	$\text{E}\left[O_{A}|X(0)=A,X(t)=B\right]=\frac{1}{\alpha +\beta }\left(\frac{\beta t-\alpha te^{-(\alpha +\beta )t}}{1-e^{-(\alpha +\beta )t}}+\frac{\alpha -\beta }{\alpha +\beta }\right)$
		$\text{E}\left[O_{B}|X(0)=A,X(t)=B\right]=t-\frac{1}{\alpha +\beta }\left(\frac{\beta t-\alpha te^{-(\alpha +\beta )t}}{1-e^{-(\alpha +\beta )t}}+\frac{\alpha -\beta }{\alpha +\beta }\right)$
B	A	$\text{E}\left[O_{A}|X(0)=B,X(t)=A\right]=\frac{1}{\alpha +\beta }\left(\frac{\beta t-\alpha te^{-(\alpha +\beta )t}}{1-e^{-(\alpha +\beta )t}}+\frac{\alpha -\beta }{\alpha +\beta }\right)$
		$\text{E}\left[O_{B}|X(0)=B,X(t)=A\right]=t-\frac{1}{\alpha +\beta }\left(\frac{\beta t-\alpha te^{-(\alpha +\beta )t}}{1-e^{-(\alpha +\beta )t}}+\frac{\alpha -\beta }{\alpha +\beta }\right)$
A	A	$\text{E}\left[O_{A}|X(0)=A,X(t)=A\right]=\frac{1}{\alpha +\beta }\left(\frac{\beta^{2}t+\frac{2\alpha \beta }{\alpha +\beta }\left(1-e^{-(\alpha +\beta )t}\right)+\alpha^{2}te^{-(\alpha +\beta )t}}{\beta +\alpha e^{-(\alpha +\beta )t}}\right)$
		$\text{E}\left[O_{B}|X(0)=A,X(t)=A\right]=t-\frac{1}{\alpha +\beta }\left(\frac{\beta^{2}t+\frac{2\alpha \beta }{\alpha +\beta }\left(1-e^{-(\alpha +\beta )t}\right)+\alpha^{2}te^{-(\alpha +\beta )t}}{\beta +\alpha e^{-(\alpha +\beta )t}}\right)$
B	B	$\text{E}\left[O_{A}|X(0)=B,X(t)=B\right]=\frac{1}{\alpha +\beta }\left(\frac{\alpha \beta t-\frac{2\alpha \beta }{\alpha +\beta }\left(1-e^{-(\alpha +\beta )t}\right)+\alpha \beta te^{-(\alpha +\beta )t}}{\alpha +\beta e^{-(\alpha +\beta )t}}\right)$
		$\text{E}\left[O_{B}|X(0)=B,X(t)=B\right]=t-\frac{1}{\alpha +\beta }\left(\frac{\alpha \beta t-\frac{2\alpha \beta }{\alpha +\beta }\left(1-e^{-(\alpha +\beta )t}\right)+\alpha \beta te^{-(\alpha +\beta )t}}{\alpha +\beta e^{-(\alpha +\beta )t}}\right)$
