# Supplementary material for: TyCHE enables time-resolved lineage tracing of heterogeneously-evolving populations
Source: bioRxiv. 2025 Oct 22:2025.10.21.683591. Preprint. [Version 1] doi: 10.1101/2025.10.21.683591 (PMC12633441; doi:10.1101/2025.10.21.683591)
Supplement: Supplement 1 [file media-1.pdf]

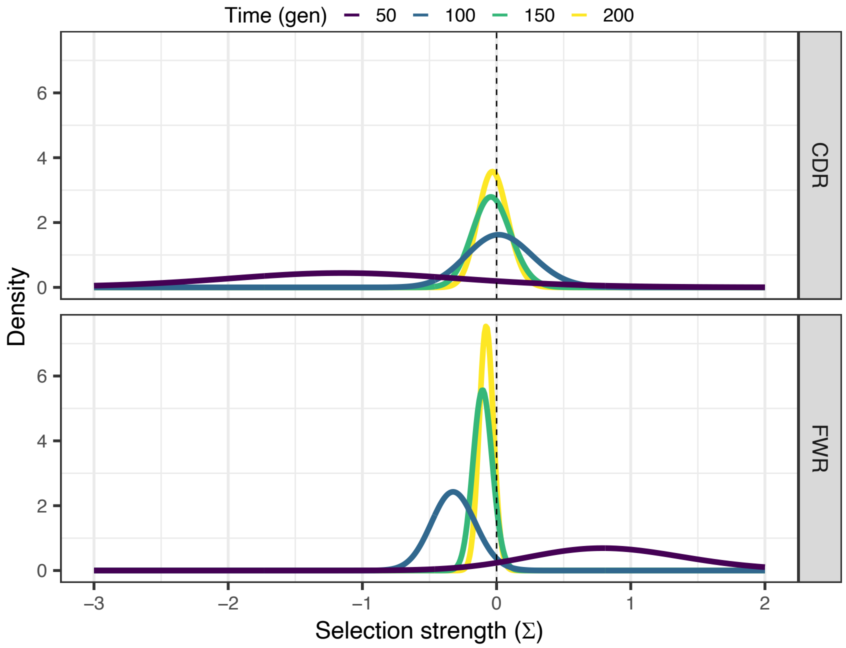

Supplemental Figure 1: BASELINe analysis of neutral simulations. Posterior distributions of selection scores from BASELINe applied to BCRs from 100 lineages sampled at 50, 100, 150, and 200 generations under simulations without selection. 5653 sequences with in-frame stop codons were removed. Sequences sampled at each generation were analyzed separately. Values below zero indicate purifying selection while values greater than zero indicate diversifying selection. CDR indicates antigen-binding complementarity-determining regions while FWR indicates structural framework regions.

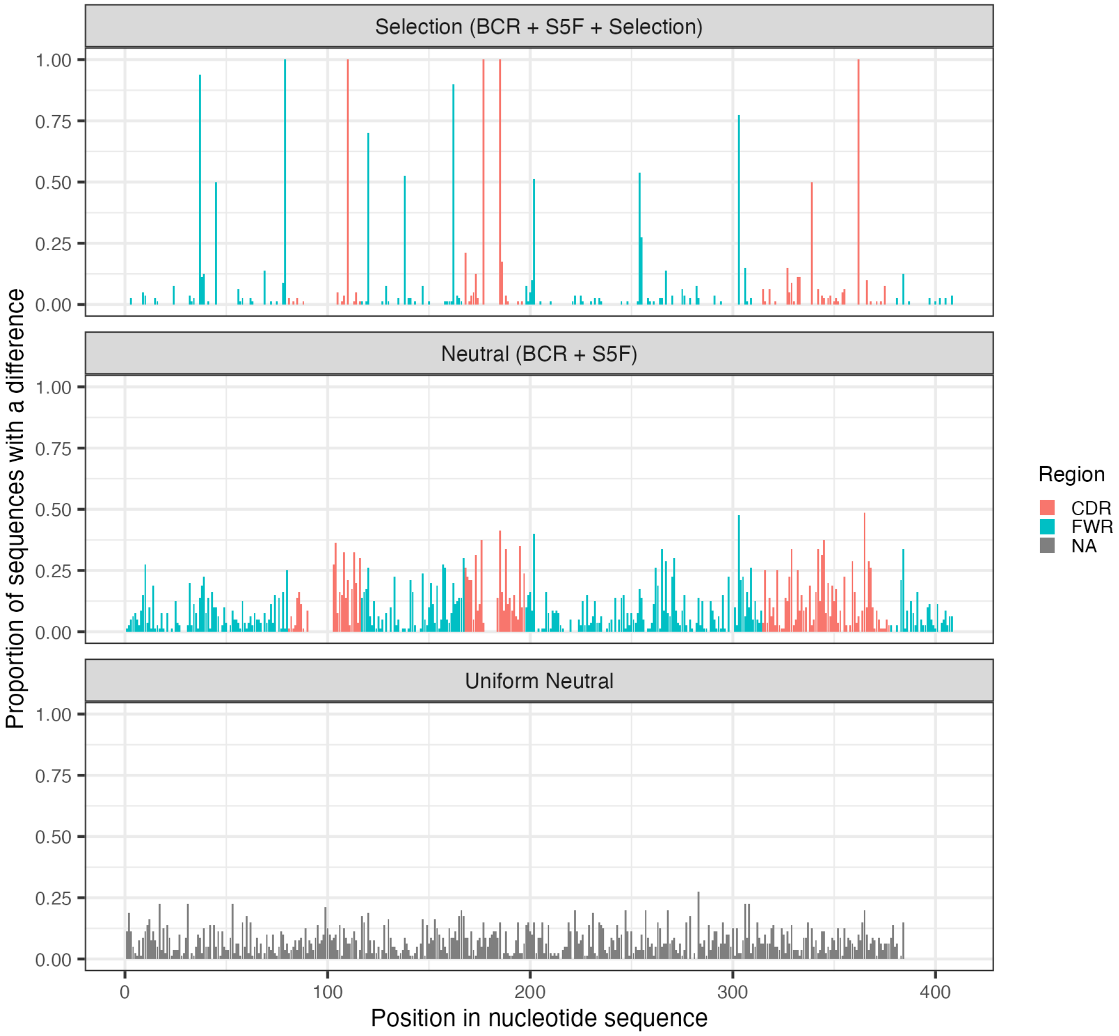

Supplemental Figure 2: Proportion of sequences with a difference at each nucleotide position for 1 clone under selection (starting BCR sequence + S5F mutation and substitution model + selection), 1 clone under BCR neutral simulation (starting BCR seq + S5F mutation and substitution model), and 1 clone under uniform neutral simulation (random starting sequence + uniform substitution probabilities). Neutral and selection models use IMGT gapped sequence position and positions are colored by region (CDR, FWR). Uniform neutral sequences have length 384 and BCR neutral and selection have un-gapped sequences of length 384.

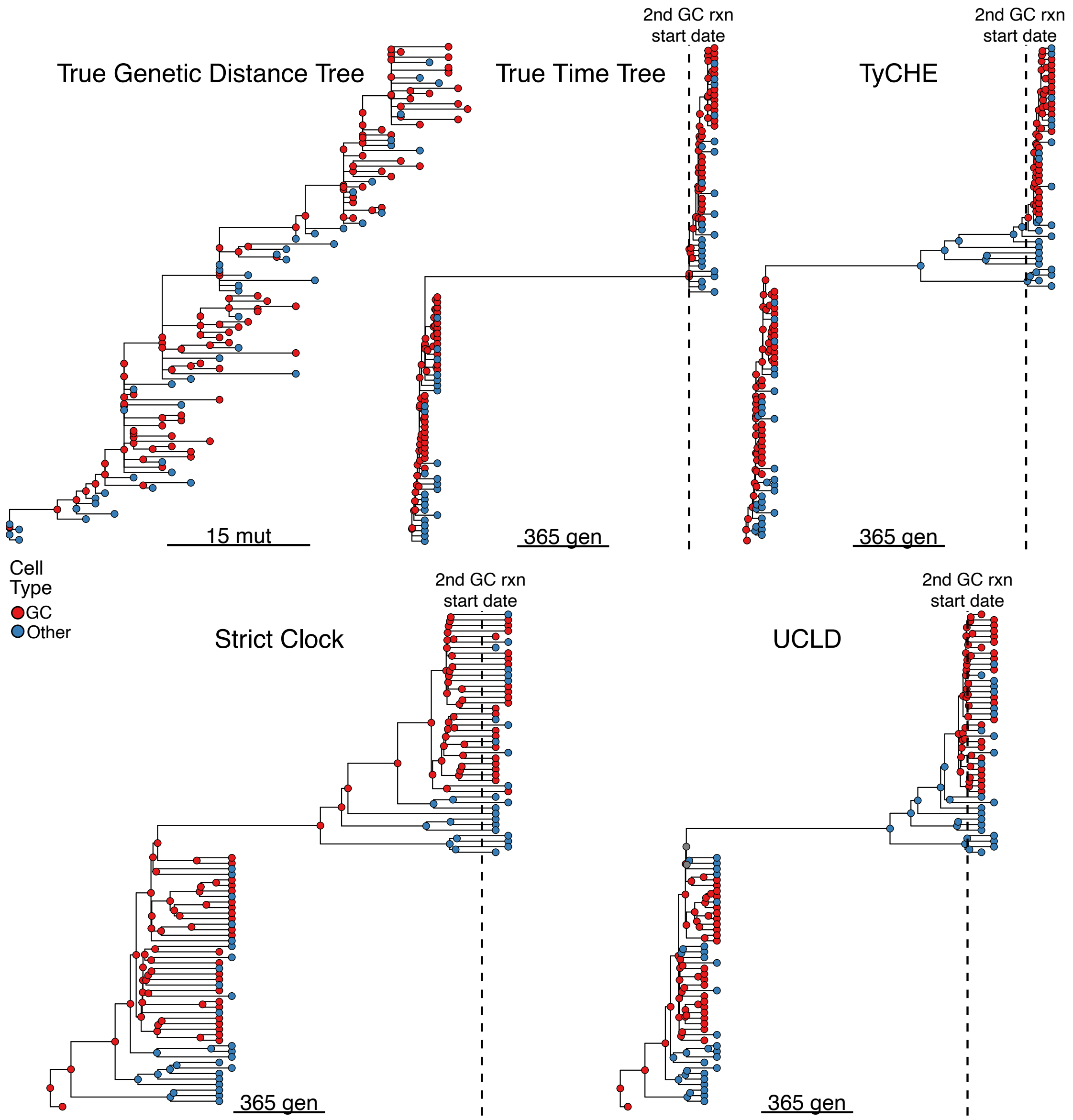

Supplemental Figure 3: Example trees from a single clonal lineage of B cells in simulated recall GC reactions. In simulations, a GC reaction occurs from generations 1-100. An unsampled memory B cell is randomly selected to exit the GC at generation 100 and wait 1000 generations before re-entering a distinct GC and beginning the secondary recall reaction, occurring from generations 1101-1200. GC and non-GC (other) B cells were evenly sampled at generations 50, 100, 150, and 200. At each time point, 24 total cells were sampled. From left to right and top to bottom, the displayed trees are the true genetic distance tree, the true time tree, the TyCHE EO with estimated clock rates estimated tree, the SC estimated tree, and the UCLD estimated tree. Dotted line refers to the true start time of the recall GC reaction; only TyCHE is able to predict that the GC reaction, measured by the date of the GC B cell MRCA of the recall GC reaction, begins after the true starting date. The UCLD predicts a recall GC reaction start date prior to the true start date, and the SC predicts a continuous GC reaction.

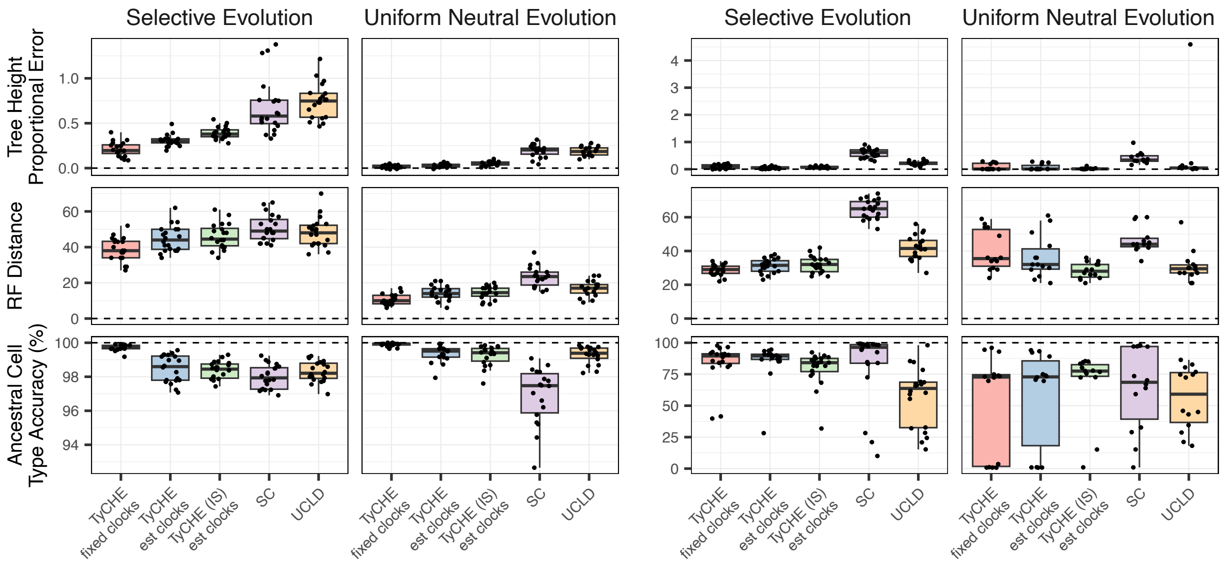

Supplemental Figure 4. Benchmarking including all converged clones. Shown are the same data as Fig. 3B-E, but without excluding one clone from GC recall simulations. Top to bottom: (*Top*) Proportional error of tree height estimates from TyCHE's EO model with fixed clock rates, EO model with estimated clock rates, IS model with estimated clock rates, as well as SC and UCLD clock models. Each dot represents one clone. (*Middle*) Robinson-Foulds distance from the estimated tree to the true tree topology. To reduce noise, branches under 5 generations long were collapsed. (*Bottom*) Mean accuracy of ancestral cell type prediction for the MRCA of each pairwise combination of tips.

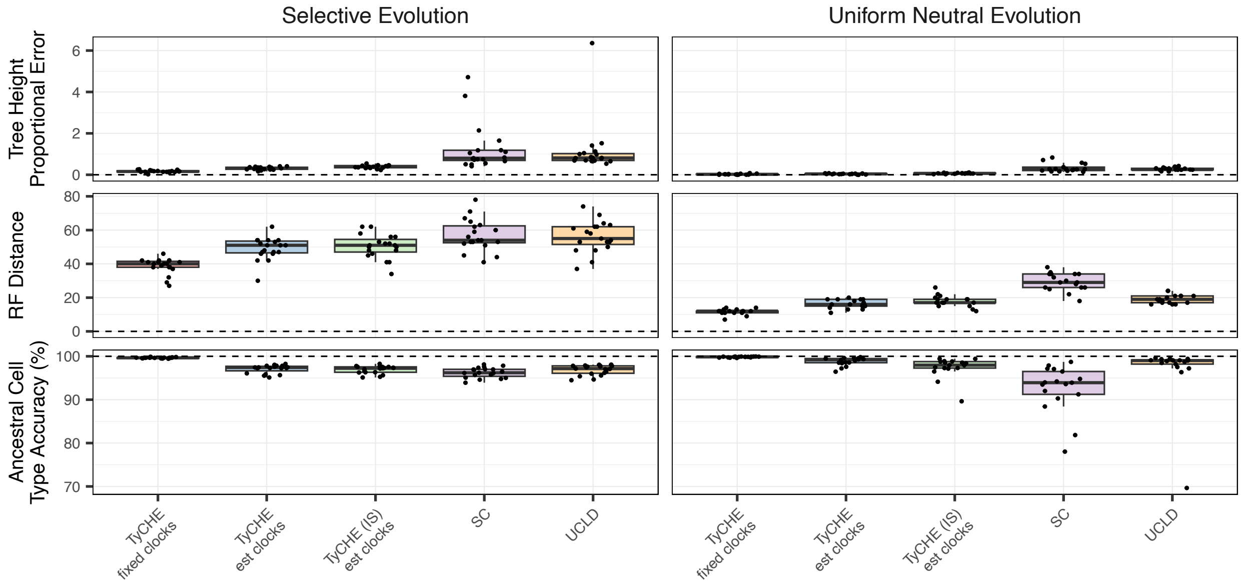

Supplemental Figure 5: Primary GC simulations with biased GC to other B cell sampling. Proportional error of tree height estimates (top), RF distance from the true tree topology (middle) and mean accuracy of ancestral cell type prediction for the MRCA of each pairwise combination of tips (bottom) from TyCHE's EO model with fixed clock rates, EO model with estimated clock rates, IS model with estimated clock rates, as well as SC and UCLD models. Sequences were simulated under either selection or uniform neutrality with a 1:3 sampling of GC:Other B cells at 50, 100, 150 and 200 generations. Each dot represents one clone.

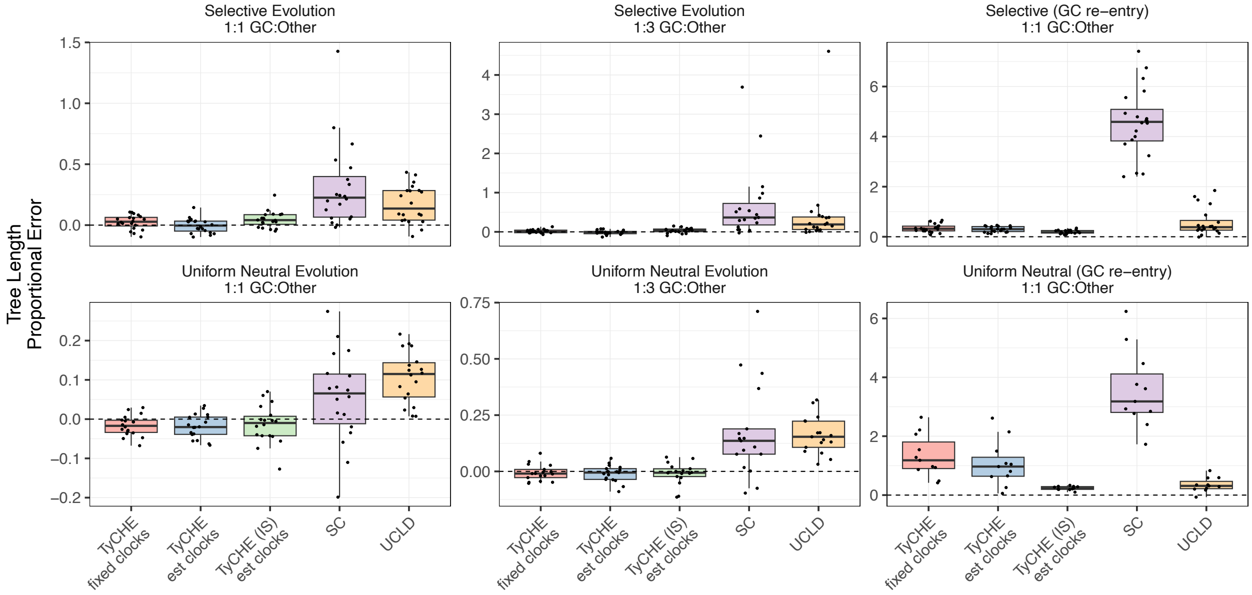

Supplemental Figure 6: Proportional error of tree length estimates from TyCHE's EO model with fixed clock rates, EO model with estimated clock rates, IS model with estimated clock rates, as well as SC and UCLD models. Each dot represents one clone.

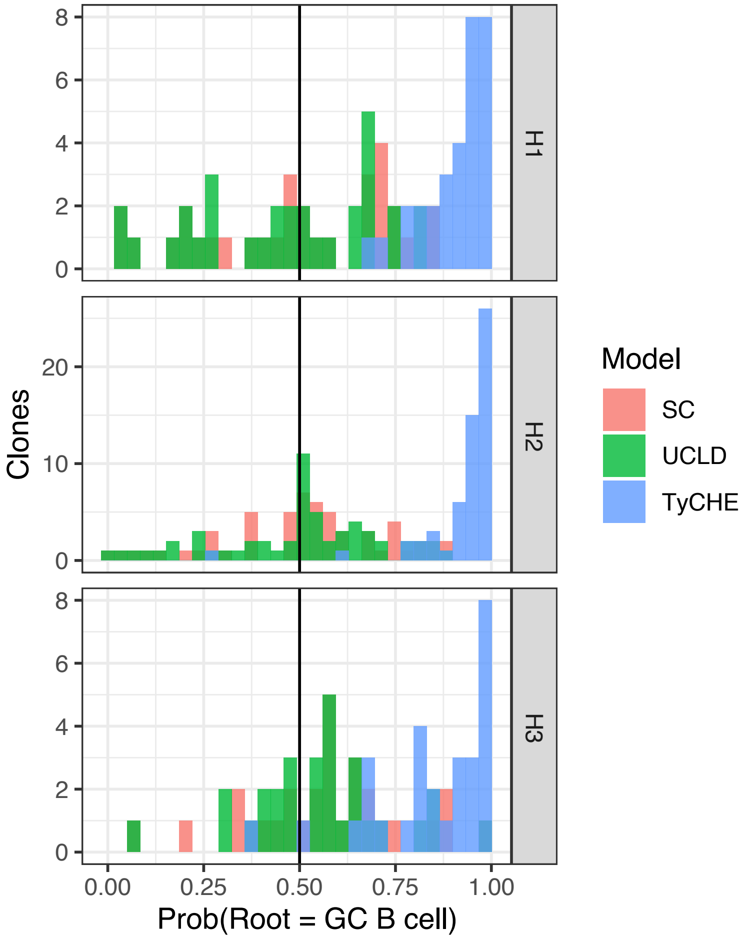

Supplemental Figure 7: Posterior probability of the root state being GC for each clone, separated by model and donor.

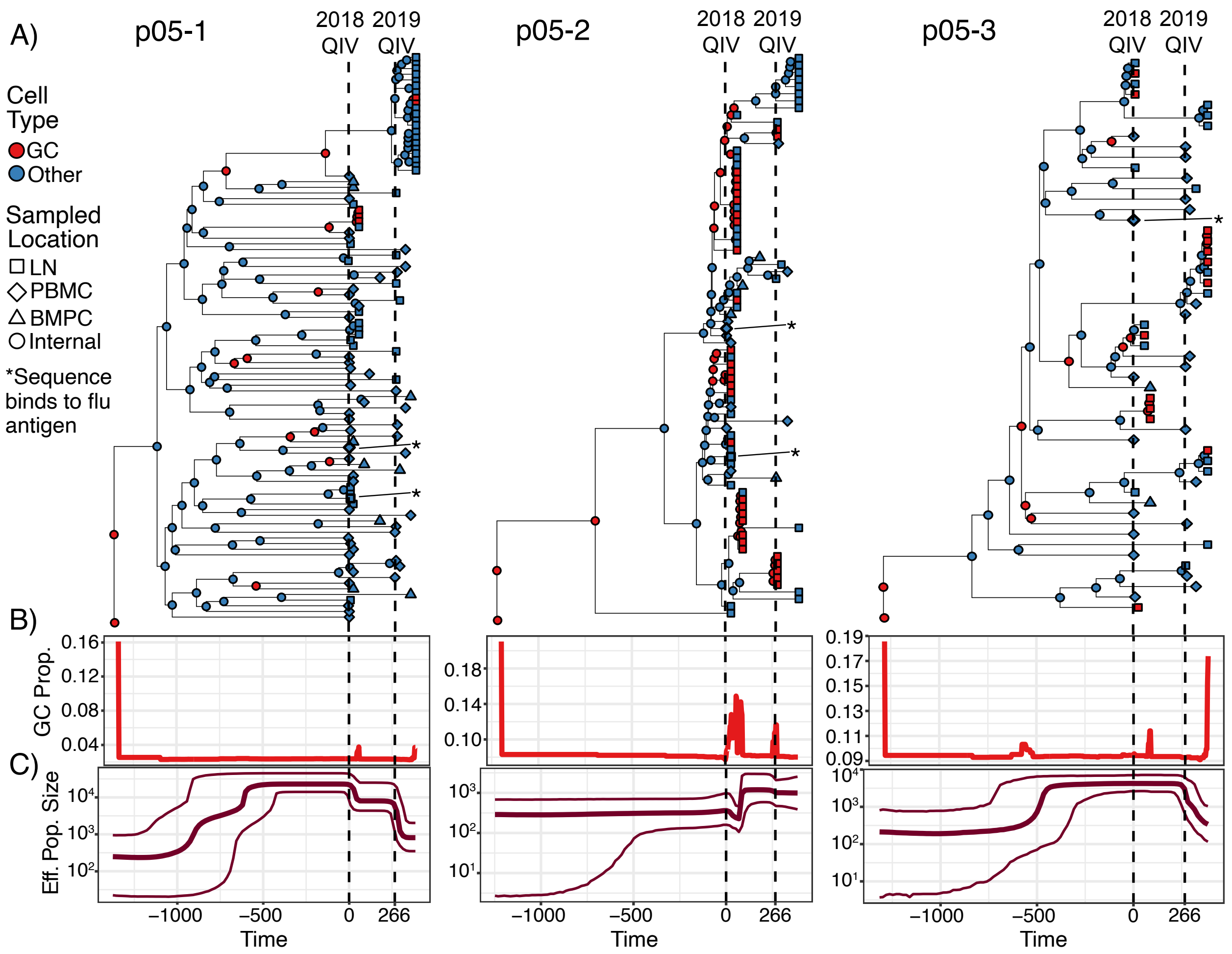

Supplemental Figure 8: Time-resolved analysis of recall GC reactions following repeated influenza vaccination of donor P05 (clones 1-3). The donor was immunized with 2018/2019 QIV vaccination at week 0, and re-immunized with 2019/2020 QIV at week 38. Samples were taken from PBMCs, lymph node (LN) fine-needle aspiration (FNA), and bone marrow plasma cells (BMPCs). **A)** TyCHE-estimated time trees. Sequences experimentally confirmed to bind influenza antigens are marked with *. Dashed lines indicate dates of influenza vaccination. **B)** Proportion of branches predicted to be GC B cells over time. **C)** Bayesian skyline plots showing changes in effective population size.

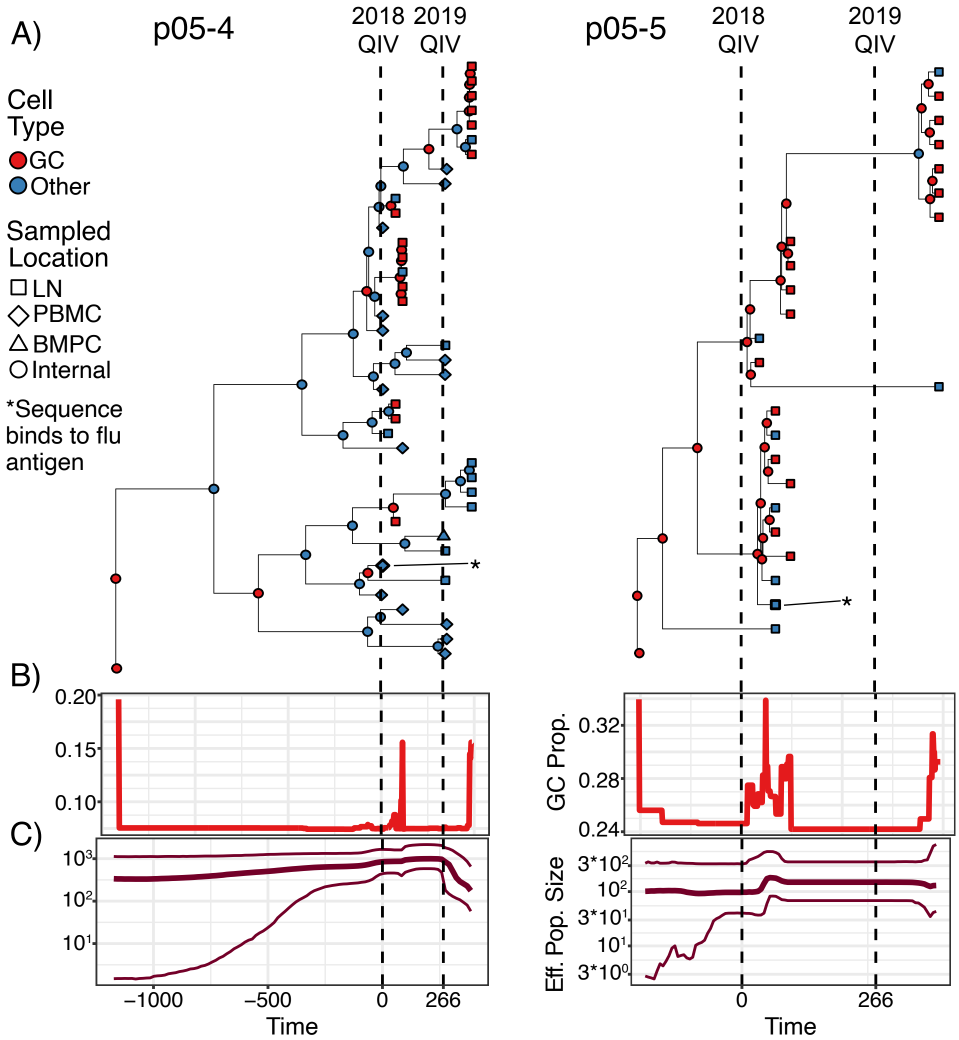

Supplemental Figure 9: Time-resolved analysis of recall GC reactions following repeated influenza vaccination of donor P05 (clones 4-5). The donor was immunized with 2018/2019 QIV vaccination at week 0, and re-immunized with 2019/2020 QIV at week 38. Samples were taken from PBMCs, lymph node (LN) fine-needle aspiration (FNA), and bone marrow plasma cells (BMPCs). **A)** TyCHE-estimated time trees. Sequences experimentally confirmed to bind influenza antigens are marked with *. Dashed lines indicate dates of influenza vaccination. **B)** Proportion of branches predicted to be GC B cells over time. **C)** Bayesian skyline plots showing changes in effective population size.

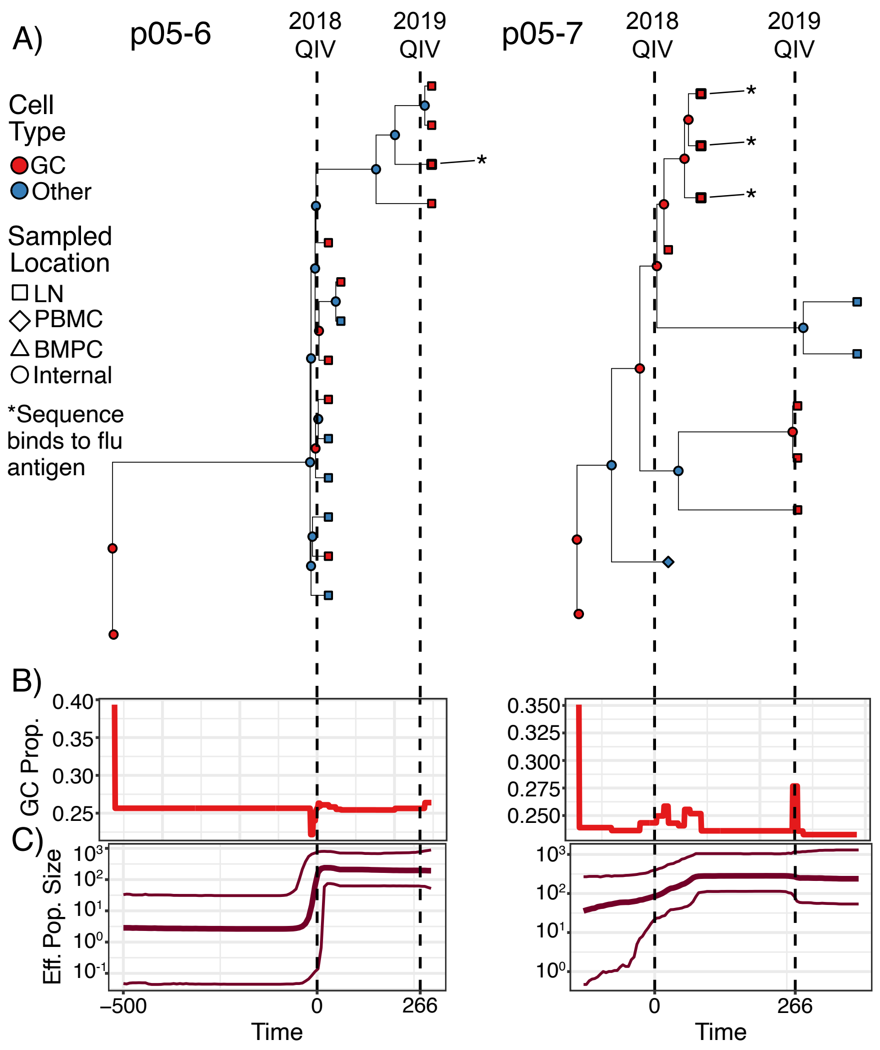

Supplemental Figure 10: Time-resolved analysis of recall GC reactions following repeated influenza vaccination of donor P05 (clones 6-7). The donor was immunized with 2018/2019 QIV vaccination at week 0, and re-immunized with 2019/2020 QIV at week 38. Samples were taken from PBMCs, lymph node (LN) fine-needle aspiration (FNA), and bone marrow plasma cells (BMPCs). **A)** TyCHE-estimated time trees. Sequences experimentally confirmed to bind influenza antigens are marked with *. Dashed lines indicate dates of influenza vaccination. **B)** Proportion of branches predicted to be GC B cells over time. **C)** Bayesian skyline plots showing changes in effective population size.
